# Effect of behavioural techniques and delivery mode on effectiveness of weight management: systematic review, meta-analysis and meta-regression

**DOI:** 10.1111/obr.12165

**Published:** 2014-03-18

**Authors:** J Hartmann-Boyce, D J Johns, S A Jebb, P Aveyard, Behavioural Weight Management Review Group

**Affiliations:** 1 Department of Primary Care Health Sciences, Radcliffe Observatory Quarter, University of OxfordOxford, UK; 2 Elsie Widdowson Laboratory, MRC Human Nutrition ResearchCambridge, UK

**Keywords:** Adult, behaviour therapy, obesity, weight loss

## Abstract

A systematic review, meta-analysis and meta-regression were conducted to evaluate the effectiveness of behavioural weight management programmes and examine how programme characteristics affect mean weight loss. Randomized controlled trials of multicomponent behavioural weight management programmes in overweight and obese adults were included. References were obtained through systematic searches of electronic databases (conducted November 2012), screening reference lists and contacting experts. Two reviewers extracted data and evaluated risk of bias. Thirty-seven studies, representing over 16,000 participants, were included. The pooled mean difference in weight loss at 12 months was −2.8 kg (95% confidence interval [CI] −3.6 to −2.1, *P* < 0.001). I^2^ indicated that 93% of the variability in outcome was due to differences in programme effectiveness. Meta-analysis showed no evidence that supervised physical activity sessions (mean difference 1.1 kg, 95% CI −2.65 to 4.79, *P* = 0.08), more frequent contact (mean difference −0.3 kg, 95% CI −0.7 to 0.2, *P* = 0.25) or in-person contact (mean difference 0.0 kg, 95% CI −1.8 to 1.8, *P* = 0.06) were related to programme effectiveness at 12 months. In meta-regression, calorie counting (−3.3 kg, 95% CI −4.6 to −2.0, *P* = 0.027), contact with a dietitian (−1.5 kg, 95% CI −2.9 to −0.2, *P* < 0.001) and use of behaviour change techniques that compare participants' behaviour with others (−1.5 kg, 95% CI −2.9 to −0.1, *P* = 0.032) were associated with greater weight loss. There was no evidence that other programme characteristics were associated with programme effectiveness. Most but not all behavioural weight management programmes are effective. Programmes that support participants to count calories or include a dietitian may be more effective, but the programme characteristics explaining success are mainly unknown.

## Introduction

Several reviews have concluded that behavioural weight management programmes (BWMPs) can be effective 1–3. However, these reviews also reveal the diversity of approaches and heterogeneity of outcomes between programmes, with some appearing highly effective and others not at all so. At first glance, it is difficult to determine reasons for these differences in effects. Most BWMPs aim for participants to lose approximately 0.5 to 1.0 kilograms (kg) per week, and involve similar diet and physical activity recommendations. However, programmes vary in the number, frequency and duration of behavioural support sessions and in the behavioural strategies used to assist participants in changing their diet and physical activity. These aspects are likely to influence the degree to which programme users change their diet and activity and hence influence weight loss. It is important to determine which aspects are associated with increased programme effectiveness, so as to maximize the potential of future interventions to achieve successful outcomes.

In recent years, a number of taxonomies identifying and characterizing specific behavioural change techniques have been developed 4–6. With the help of these taxonomies, interventions can be broken down into active ingredients that can be categorized, and evaluations can progress from simply treating complex interventions as a uniform group. Specifying the behavioural components of a programme improves clarity of description, aids those who plan to implement the intervention and helps with the identification of programme characteristics which contribute to effectiveness. The CALO-RE taxonomy, a list of 40 behavioural change techniques used to help people change their physical activity and eating behaviours, is particularly relevant to the analysis of BWMPs 5.

This review aims to evaluate the effectiveness of multicomponent BWMPs, to systematically apply behavioural change taxonomy to characterize the interventions used in randomized controlled trials of BWMPs and to examine which characteristics of programme delivery are associated with programme effectiveness. These characteristics relate to: delivery of diet components, delivery of physical activity components, intervention format and behavioural change techniques.

## Methods

### Search and inclusion criteria

A review protocol was agreed prior to commencing work (Supporting Information File S1). We searched BIOSIS, the Cochrane Database of Systematic Reviews, CENTRAL, the Conference Proceedings Citation Index, the Database of Abstracts of Reviews and Effects, EMBASE, the Health Technology Assessment database, MEDLINE, PsycINFO and the Science Citation Index between inception and November 2012 for randomized and quasi-randomized controlled trials, using terms for overweight and obesity, diet and physical activity and weight loss interventions. The search was based on a comprehensive review and search undertaken by Loveman *et al*. and hence references prior to May 2009 were obtained by screening search results reported in that review (Supporting Information File S2) 2. Additional studies were obtained from screening relevant systematic reviews. We also contacted experts in the field to enquire about studies our searches may have missed (e.g. unpublished).

To be included, studies had to have recruited adults (≥18 years) with a body mass index (BMI) of ≥25 kg m^−2^ (or a BMI of ≥23 kg m^−2^ in Asian populations). Interventions had to involve multiple contacts with the provider (or multiple web sessions if an internet intervention) and be clearly defined multicomponent weight management programmes, i.e. contain diet, physical activity and behaviour change techniques with a sufficiently detailed description of each component.

We excluded studies in pregnant women, people with eating disorders and those where weight loss was used as a treatment for a medical condition such as diabetes. We also excluded programmes that involved surgery or medication or incorporated other lifestyle changes, e.g. smoking cessation.

Studies were required to include a measure of weight change at 12 months or greater from baseline. To be included, trials had to have a non-BWMP control arm (ranging from self-help material to contact with someone without specific training in weight management) or provide a direct comparison of multicomponent BWMPs based on a variable we planned to investigate (see Table 1).

**Table 1:** Delivery variables investigated

Variable	Evaluated as
Delivery of diet components	
Participant asked to monitor energy intake (referred to as calorie counting in remainder of report)	Binary: yes/no
Dietary programme delivered at least in part by a dietitian	Binary: yes/no
Delivery of physical activity components*	
Advice supported by supervised activity sessions	Binary: yes/no
Advice required specialized equipment or setting to enact	Binary: yes/no
Intervention format	
Delivery method	Categorical: group/individual/both
Length of intervention up to 12 months	Continuous: months
Contact type	Binary: face-to-face/remote
Number of sessions offered in the first 12 months of a programme	Continuous
Frequency of contact	Continuous: in indirect comparisons, number of weeks between contacts in most intensive phase; in direct comparisons, more versus less contacts over a set period of time
Participant given weight loss goal	Binary: yes/no
Use of follow-up prompts i.e. does contact frequency decline over time?	Binary: yes/no
Behavioural change techniques used (see Table 2 for subcategories)	Ordinal: by domain

* Note, we did not include person delivering the physical activity as a variable due to inconsistencies in reporting regarding the qualifications of those delivering the physical activity components of the intervention.

**Table 2:** Index to domains of taxonomy items

Domain	Taxonomy item*
Goals and planning	05- Goal setting (behaviour)
	06- Goal setting (outcome)
	07- Action planning
	08- Barrier identification/problem solving
	10- Prompt review of behavioural goals
	11- Prompt review of outcome goals
	20- Provide information on where and when to perform the behaviour
	25- Agree behavioural contract
	35- Relapse prevention/coping planning
Reward and threat	12- Prompt rewards contingent on effort or progress towards behaviour
	13- Provide rewards contingent on successful behaviour
	14- Shaping
	32- Fear arousal
	40- Stimulate anticipation of future rewards
Regulation	36- Stress management/emotional control training
	38- Time management
Antecedents	24- Environmental restructuring
Identity	30- Prompt identification as role model/position advocate
Self-belief	18- Prompting focus on past success
	33- Prompt self-talk
Covert learning	34- Prompt use of imagery
Feedback and monitoring	16- Prompt self-monitoring of behaviour
	17- Prompt self-monitoring of behavioural outcome
	19- Provide feedback on performance
Social support	29- Plan social support/social change
	37- Motivational interviewing
	39- General communication skills training
Shaping knowledge	21- Provide instruction on how to perform the behaviour
Natural consequences	01- Provide information on consequences of behaviour in general
	02- Provide information on consequences of behaviour to the individual
	31- Prompt anticipated regret
Comparison of behaviour	03- Provide information about others' approval
	04- Provide normative information about others' behaviour
	22- Model/demonstrate the behaviour
	28- Facilitate social comparison
Associations	23- Teach to use prompts/cues
Repetition and substitution	09- Set graded tasks
	15- Prompting generalization of a target behaviour
	26- Prompt practice

* Number refers to original number in CALO-RE taxonomy.

### Data collection and outcome measurement

Titles and abstracts were assessed by a single reviewer with a sample checked by a second. Data extraction was completed independently by two reviewers with discrepancies resolved by discussion or by referral to a third reviewer. Two reviewers assessed each study for risk of bias based on criteria developed by the York Centre for Reviews and Dissemination 7, including random sequence generation, allocation concealment, attrition and selective reporting. If further detail was required on any aspect of study design or outcome, we sought related publications and treatment protocols and contacted study authors.

Alongside data extraction, two reviewers coded each programme against a checklist of techniques from the CALO-RE taxonomy 5. Programmes were coded as ‘yes’, ‘no’ or ‘unclear’ for use of each behavioural change technique. The ‘unclear’ code was applied where a technique was not explicitly stated, but reviewers agreed elements of the programme description implied that it was used.

Our aim was to assess whether the methods used to deliver BWMPs were related to programme effectiveness. The key variables relating to delivery methods are listed in Table 1.

Due to the large number of behaviour change techniques and relatively small number of included studies, we clustered taxonomy items into domains of techniques (Table 2) to allow analysis. Domains were based on previously published categories 6. For example, the techniques ‘prompt self-monitoring of behaviour’, ‘prompt self-monitoring of behavioural outcome’ and ‘provide feedback on performance’ were all included in the ‘feedback and monitoring’ domain. Each intervention received a score for each domain, reflecting the number of techniques from that domain used in the intervention (yes = 1; unclear = 0.5; no = 0). One item from the CALO-RE taxonomy, ‘use of follow-up prompts’, is not included in the behaviour change domains as it was assessed as an individual variable.

The primary outcome was mean weight change calculated using baseline observation carried forward (BOCF), an intention-to-treat analysis which imputes baseline weight for participants missing at follow-up. Where data were not reported in this form, BOCF was calculated using complete case data as described previously 8. Where we were unable to obtain complete case data, we calculated BOCF using the data reported in last observation carried forward or other intention-to-treat models. Where studies did not report data at 12 months, weight change at 13 to 18 months was used in its place. One small study was excluded from statistical analyses because of insufficient information from which to calculate BOCF weight change 9.

As variation in the instructions given to participants could impact programme effectiveness, for example restricting energy intake to a greater extent, we also assessed the content of the dietary and activity instructions. We extracted data on the daily energy targets in kcal (which is separate from asking participants to count calories), the nutritional composition of the diets prescribed, and the weekly physical activity targets at programme end, as these were usually incremental.

### Statistical analysis

This proceeded in three steps. Firstly, we meta-analysed all studies in which the effectiveness of a BWMP was compared with a non-BWMP control to confirm heterogeneity of outcome and to quantify statistical heterogeneity. Secondly, we meta-analysed trials in which participants received a BWMP in both arms but were randomized to receive this with and without a particular component of interest, such as randomized to supervised physical activity or activity counselling only. Thirdly, as such direct comparisons were few, we proceeded to conduct meta-regression across trials comparing BWMPs with control, comparing effect sizes in BWMPs that included a pre-specified characteristic of interest to effect sizes in those that did not.

In the first and second steps, random effects meta-analyses were conducted in Review Manager 5.2 (The Nordic Cochrane Centre, Copenhagen, Denmark) to examine mean difference in weight change at 12 months 10. Pooled results are presented as mean differences (kg) with 95% confidence intervals (CI). The I^2^ statistic and 95% prediction intervals are used to present statistical heterogeneity between studies 11,12. Where a study contributed more than one intervention arm to an analysis, we split the control group equally to avoid double counting in the pooled result. We used a funnel plot to investigate possible publication bias in comparisons of 10 or more studies.

In the third step, random effects meta-regression was conducted using STATA v12 (Stata Statistical Software: Release 12; StataCorp LP, College Station, TX, USA) for all studies with a non-BWMP control arm. We ran univariate models to examine the association between study effect size and mode of delivery (delivery of diet components, delivery of physical activity components and intervention format) and, separately, the behavioural techniques used. We ran multivariable models adding the variable with the strongest association in univariate analysis first and adding all others in turn, regardless of significance in the univariate model. These were retained in the model if they were statistically significant (*P* < 0.05), building the model in steps until no further variables were significant. Where a behavioural change technique domain was significantly associated with weight change, we ran exploratory meta-regressions on the techniques within that cluster. Insufficient data were available to statistically assess potential confounding from the diet and physical activity targets reported, and hence we summarize these narratively in the text.

## Results

After removing duplicates, the search retrieved 2,210 references in total, the majority from database searches (Supporting Information Fig. S1). After excluding 2004 references based on title and abstract, full text was retrieved and screened for 206 references. Of these, 153 were excluded, with the most common reason being study design. Fifty-three references met our inclusion criteria, representing 37 studies. Thirty studies included a non-BWMP control, 29 of which had sufficient outcome data to be included in the meta-regression, representing 40 intervention versus control comparisons. Ten studies directly compared BWMPs on a variable of interest.

### Characteristics of included studies

The 37 included studies represent over 16,000 participants, with 13,453 included in the primary meta-analysis and the meta-regression. The number of participants in each study ranged from 65 to over 2,100, with a mean of 378 participants per study. The mean age of study participants ranged from 32 to 70 years. The majority of participants were female (68% average). Six studies recruited women only and two recruited men only. Over half (53%) of all studies were conducted in the US. Intervention characteristics are summarized below. Supporting Information Table S1 provides further detail on included studies.

#### Delivery of diet components

Of the 40 intervention versus control comparisons, 16 interventions involved some participant contact with a dietitian (ranging from part of one session to multiple contacts). Just over half (*n* = 21) of the interventions asked participants to monitor their own energy intake in order to meet set calorie goals.

#### Delivery of physical activity components

Of the 40 interventions versus control comparisons, 16 interventions provided supervised physical activity sessions. Two studies provided direct comparisons of supervised versus recommended physical activity. Six interventions required a specific setting or type of equipment to perform the physical activity components.

#### Intervention format

On average, interventions were 18 months long, ranging from 3 months to 3 years. However, in this review, outcome was assessed at 12 months and therefore both the median and the maximum intervention length of the programme analysed was 12 months. The total number of sessions ranged from two to 216, median 39. Contact frequency decreased in intensity over time in 16 interventions. Twelve interventions were delivered in both group and individual sessions, 16 were delivered via group sessions only and 21 were delivered via individual sessions only. Five interventions did not involve in-person contact. Six studies directly compared more versus less contact and three directly compared in-person versus remote contact only.

#### Behavioural change techniques

**Figure 1:**
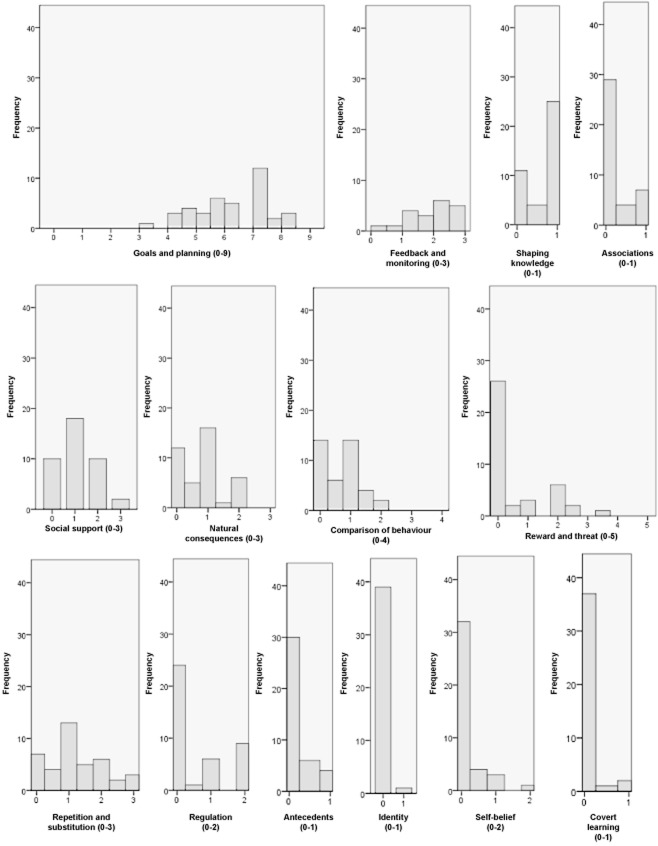
Histogram of behaviour change technique domain scores for interventions contributing to meta-regression.

Interventions appeared to be very similar in terms of the behaviour change techniques used, despite large variation in the detail with which programme components were reported. Consequently, the scores representing the total number of techniques used in each domain were similar between interventions. For the most part, interventions scored highly in ‘goals and planning’ and ‘feedback and monitoring’, and lower in other domains. Figure 1 shows the number of interventions with each score within the domains. The majority of interventions included: goal setting and review of goals, action planning, barrier identification and/or problem solving, graded tasks (tasks increase in intensity over time), self-monitoring of behaviour, feedback on performance, instruction on how to perform behaviour and planning social support and/or social change.

#### Potential confounding factors

Aside from the methods used to change participants' behaviour, the nature of the diet and the amount of physical activity prescribed could influence programme effectiveness and confound the assessment of the influence of programme characteristics on weight loss. Most studies reported the nature of the advice given in minimal detail, often implying that it was ‘standard’.

In the 27 interventions where diet composition was reported, 25 reported or implied low fat diets were recommended. Energy targets were not reported in sufficient detail to allow us to control for them statistically, but where reported, the majority were calculated based on an individual's weight at baseline and, most commonly, aimed for a deficit of 2,090 kiloJoules (kJ) per day (500 kilocalories [kcal]). Sixteen interventions described their energy prescription in terms of the weekly weight loss they sought to achieve: 11 aimed for 1.0 kg per week, with the others ranging from 0.3 kg to 2.0 kg. Overall, the energy prescriptions and the nature of the diets differed only modestly between programmes.

End of programme weekly physical activity targets were available for 36 of the 40 interventions included in the meta-regression, although limited reporting meant we were unable to calculate a standard metric for recommended physical activity across interventions. Where reported, interventions prescribed three to seven physical activity sessions weekly (average five) of moderate or vigorous intensity. Targets ranged from 150 min to 360 min of physical activity a week, with a mean of 190 min.

### Risk of bias

Fourteen of the 37 included studies were judged to be at low risk of bias across all areas assessed. Randomization and allocation procedures were judged to place results at risk of selection bias in three studies, and in a further 15 they were not described in sufficient detail and judged to be unclear. Nine studies were judged to be at high risk of bias for selective reporting because they did not report some outcomes which authors had prespecified. Four studies were judged to be at high risk of attrition bias because of large dropout across all arms (>50%) or uneven dropout between arms (difference of >20%). Supporting Information Table S2 shows risk of bias judgements for each included study. Sensitivity analysis did not detect a significant difference in weight loss between studies at low risk of bias and studies at unclear or high risk of bias. A funnel plot did not suggest the presence of publication bias in the group of studies comparing a BWMP with control. There were insufficient studies to create funnel plots for other comparisons.

### Weight loss

**Figure 2:**
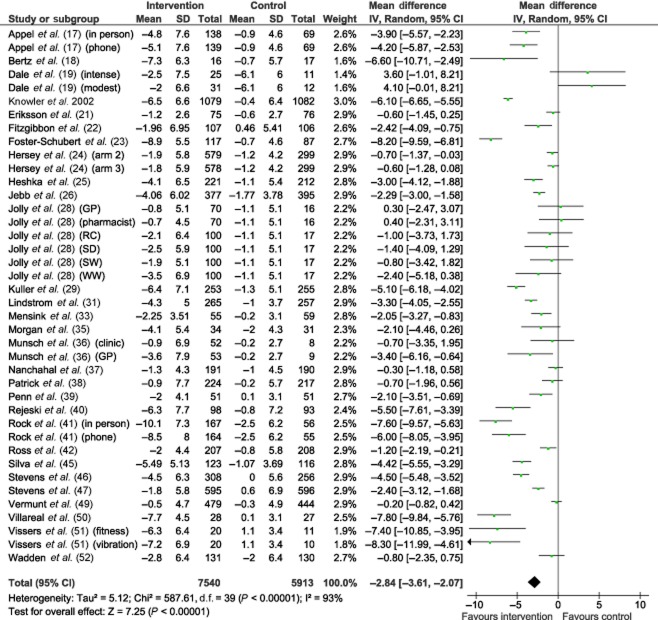
Meta-analysis of mean difference in weight loss at 12 months, intervention versus non-behavioural weight management programme control. GP, general practice; RC, Rosemary Conley; SD NHS, size down; SW, slimming world; WW, weight watchers.

The pooled mean weight loss for all intervention versus control comparisons was 2.8 kg (95% CI −3.6 to −2.1, Fig. 2). However, the estimates were highly variable, with an I^2^ value of 93% suggesting that 93% of the variability between studies was due to differences in effectiveness and 7% due to sampling variation. The 95% prediction interval indicated that 95% of programme effectiveness estimates would lie between a weight loss of 7.5 kg and weight gain of 1.8 kg.

#### Direct comparisons of intervention characteristics

##### Delivery of diet components

There were no direct comparisons for the components of diet delivery that we planned to evaluate.

##### Delivery of physical activity components

**Figure 3:**
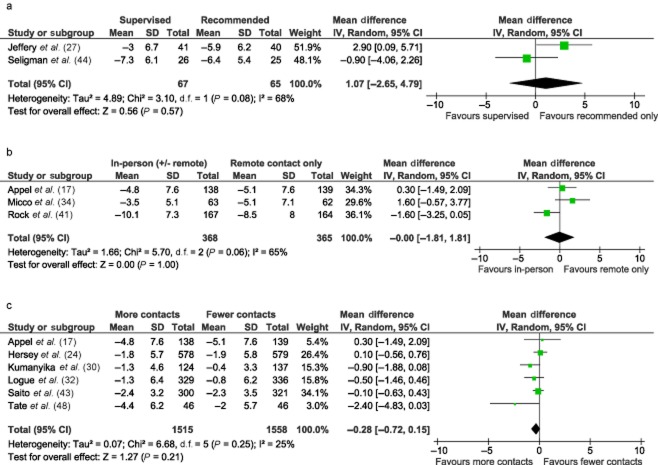
Meta-analysis of direct comparisons. (a) Supervised physical activity sessions versus recommended physical activity only. Weight loss at 12 months. (b) Some in-person contact versus remote contact only. Weight loss at 12 months. (c) More versus less contact over a set period of time. Weight loss at 12 months.

Pooled results from two studies comparing supervised physical activity sessions with recommended physical activity only did not detect a significant difference in weight change at 12 months (mean difference +1.1 kg, 95% CI −2.7 to +4.8), although statistical heterogeneity was high (I^2^ = 68%) (Fig. 3). There were no direct comparisons based on whether or not the physical activity programme required special resources to enact.

##### Intervention format

Six studies compared more versus less contact over a fixed period of time. Pooled results did not detect a significant difference in weight change at 12 months (mean difference −0.3 kg, 95% CI −0.7 to +0.2, I^2^ = 25%). Pooled results from three studies randomizing participants to in-person or remote contact only also did not detect a significant effect (mean difference −0.0 kg, 95% CI −1.8 to +1.8), although statistical heterogeneity was high (I^2^ = 65%). There were no direct comparisons for length of intervention, group versus in-person delivery, calorie counting or use of follow-up prompts.

##### Behavioural change techniques

No studies provided direct comparisons based on the use of specific behavioural change techniques.

#### Indirect comparisons of intervention characteristics (meta-regression)

##### Diet, physical activity and intervention format

In univariate analysis, longer programmes and programmes in which participants were asked to count calories were significantly associated with greater weight loss, while providing more sessions was associated with significantly lower weight loss (Table 3). Calorie counting had the strongest association with weight loss and, retaining this in the model, length of intervention, number of sessions and involvement of a dietitian were all significantly associated with greater weight loss at 12 months when added individually. Following the stepwise approach, the models were rerun including both calorie counting and involving a dietitian in delivery (the strongest association of the three). The association with weight loss at 12 months remained significant for calorie counting (−3.3 kg, 95% CI −4.6 to −2.0) and dietitian involvement (−1.5 kg, 95% CI −2.9 to −0.2). No other significant associations were detected with these two variables in the model.

**Table 3:** Results from meta-regressions

Component	Univariate coefficient (95% CI, *P* value)	Multivariable coefficient† (95% CI, *P* value)
Delivery of diet components		
Contact with dietitian	−1.0 kg (95% CI −2.8 to +0.8, *P* = 0.26)	−1.5 kg, 95% CI −2.9 to −0.2, *P* < 0.001
Calorie counting	−3.3 kg (95% CI −4.7 to −1.9, *P* < 0.001)	−3.3 kg, 95% CI −4.6 to −2.0, *P* = 0.027
Delivery of physical activity components		
Supervised physical activity*	−1.7 kg (95% CI −3.5 to 0.0, *P* = 0.055)	
Specific equipment or setting required for physical activity	−0.8 kg (95% CI −3.4 to +1.9, *P* = 0.56)	
Intervention format		
Group and individual contact	−0.4 kg (95% CI −1.6 to +2.7, *P* = 0.68)	
Individual contact only	−0.04 kg (95% CI −1.9 to +2.0, *P* = 0.97)	
Group contact only	0.4 kg (95% CI −1.6 to +2.3, *P* = 0.71)	
Face-to-face contact*	−0.6 kg (95% CI −3.2 to +2.1, *P* = 0.66)	
Programme length (up to 12 months)	−0.3 kg (95% CI 0.5 to −0.1, *P* = 0.009)	
Contact frequency (defined as average number of weeks between contacts)*	0.1 kg (95% CI −0.3 to +0.5, *P* = 0.60)	
Number of sessions of therapy	+0.03 kg (95% CI −0.04 to −0.01, *P* = 0.004)	
Decreasing intensity of support	−1.4 kg (95% CI −3.0 to +0.2, *P* = 0.092)	
Behavioural change techniques (by domain)		
Goals and planning	−0.4 kg (95% CI −1.1 to +0.2, *P* = 0.18)	
Comparison of behaviour	−1.5 kg (95% CI −2.9 to −0.1, *P* = 0.032)	−1.5 kg (95% CI −2.9 to −0.1, *P* = 0.032)
Self-belief	+2.1 kg (95% CI +0.1 to +4.1, *P* = 0.040)	
Shaping knowledge	−1.7 kg (95% CI −7.7 to +0.2, *P* = 0.082)	
Repetition and substitution	−0.9 kg (95% CI −1.9 to +0.1, *P* = 0.081)	
Antecedents	−1.2 kg (95% CI −3.8 to +1.5, *P* = 0.38)	
Feedback and monito ring	−0.4 kg (95% CI −1.5 to +0.7, *P* = 0.47)	
Social support	+0.5 kg (95% CI −0.6 to +1.6, *P* = 0.36)	
Covert learning	−0.3 kg (95% CI −4.2 to +3.5, *P* = 0.87)	
Reward and threat	+0.6 kg (95% CI −0.3 to +1.5, *P* = 0.19)	
Regulation	+1.0 kg (95% CI −0.04 to +2.0, *P* = 0.060)	
Associations	+0.3 kg (95% CI −2.1 to +2.6, *P* = 0.82)	
Natural consequences	+1.1 kg (95% CI −0.2 to +2.5, *P* = 0.091)	
Identity	+2.1 (95% CI −4.0 to +8.2, *P* = 0.49)	

* Consistent with direct comparisons.

† Where variable included in final model.

##### Behavioural change techniques

In a univariate model, each additional technique within the ‘comparison of behaviour’ domain was associated with an additional 1.5 kg weight loss at 12 months (95% CI −2.9 to −0.1). This domain is based on techniques that compare an individual's behaviour with others, and includes four techniques: ‘provide information about others' approval’, ‘provide normative information about others' behaviour’, ‘model/demonstrate the behaviour’ and ‘facilitate social comparison’ (a technique which involves explicitly drawing attention to others' performance to elicit comparisons). An exploratory analysis of the individual techniques within this domain showed that only ‘model/demonstrate behaviour’ was significantly associated with weight loss when controlling for the other three techniques. Use of this technique was associated with 2.7 kg greater weight loss at 12 months (95% CI −4.5 to −0.8 kg).

Conversely, the greater use of self-belief techniques was associated with lower effectiveness in a univariate model (coefficient +2.1 kg, 95% CI +0.1 to +4.1). An exploratory meta-regression of the individual techniques within this domain (‘prompting focus on past success’ and ‘prompting self-talk’) did not detect a significant association of either individual technique with weight change. No other domains of techniques were significantly associated with weight change at 12 months.

Each domain was included in a multivariable model controlling for the effect of the most significant variable, ‘comparison of behaviour’. With this variable in the model, no other variable was significantly associated with weight loss.

## Discussion

Weight change at 1 year varied substantially between programmes, but the reasons for this remain largely unclear. Meta-analysis of trials showed no evidence that more frequent contact with a therapist within a set period led to greater weight loss, and the precision of the estimate excluded any clinically significant effect of more frequent contact. Delivering programmes in-person versus remotely and providing supervised physical activity sessions also showed no evidence of greater benefit, but the data were too imprecise and heterogeneous to draw conclusions. Meta-regression showed that programmes that asked participants to count calories and provided at least some contact with a dietitian were associated with greater weight loss, as were programmes that facilitated social comparison.

To our knowledge, this review is the first to use both direct and indirect comparisons to examine whether the format and content of BMWPs are associated with weight change. A similar review by Dombrowski *et al*. using only indirect comparisons detected a significant association between contact frequency and weight loss 13. We did not detect an effect of higher contact frequency in direct comparisons (nor did we detect an association in meta-regression). Findings from direct comparisons are more robust as they are less prone to detecting spurious associations. Dombrowski *et al*. also found that programmes using the behaviour change techniques of providing instructions, self-monitoring, relapse prevention and prompting practice (prompting participant to rehearse/repeat behaviour) were associated with greater weight loss, whereas our review did not detect any significant associations between these variables and weight change. Unlike Dombrowski *et al*., we restricted our outcome to weight change at 12 months and reanalysed data so that loss to follow-up was accounted for in the same manner in all studies, removing a possible spurious cause of apparent differences in effectiveness across studies. In addition, Dombrowski *et al*. restricted inclusion to trials in older people with higher BMI and comorbidity related to obesity, did not require interventions to be multicomponent, and used an earlier, more general behavioural taxonomy 4. These methodological differences may explain the differences in findings.

In our analysis, only one of the 14 domains of behavioural techniques was significantly associated with effectiveness (see Table 2 for a complete list of domains). This finding could be due to chance given the number of comparisons, but the overall apparent lack of impact of behavioural change techniques is at first sight puzzling. However, there was a striking homogeneity in the behavioural techniques described in published reports, which limited the ability of statistical analysis to identify associations. For example, all but two interventions involved self-monitoring, a technique previously associated with increased effectiveness 14,15. Our ability to detect differences in effectiveness between programmes with different behavioural techniques may have also been restricted by the assumption that the ‘dose’ of technique in each domain was proportional to the number of techniques used and by limited reporting. Fidelity assessments that record what techniques are used in delivery of the programme would be useful, as investigations of other behavioural interventions have found important differences between what was specified and what was delivered 16. No trials in this review reported fidelity of delivery of behavioural components.

Our review findings on the association of weight loss with dietitian involvement, calorie counting and comparison of behaviour techniques come from cross-study comparisons. These interventions differed in other ways than simply the programme characteristics we investigated in the meta-regression, as did the participants involved. This could create spurious associations or mask true differences in effectiveness related to other aspects of programme delivery. However, had other characteristics of programme delivery had important influences on weight change they would have been controlled in meta-regression, although of course we cannot exclude residual confounding. While limited reporting meant we were unable to statistically evaluate potential confounding based on the specifics of diet and physical activity targets, the similarities in available data suggest that these were unlikely to significantly confound our results. It might be supposed that the detail recorded regarding the behavioural intervention has increased over time and indeed we found that more recent studies reported more components from the taxonomy. However, we found no evidence of an association between publication date and weight change. Clearer reporting of the characteristics of BWMPs, including diet, activity and behavioural components, would enable future analyses to more rigorously control for potential sources of confounding.

Like most systematic reviews, summarizing and comparing data was difficult because of insufficient detail and inconsistent descriptions between studies. We make the following recommendations for future studies of this type. It would help if the field could agree on presenting data on weight loss accounting for people lost to follow-up. We converted all weights presented to BOCF to account for loss to follow-up, which reduced spurious heterogeneity among studies, which had used several ways and none to account for loss. If future studies reported BOCF outcomes either as primary outcomes or sensitivity analyses, it would facilitate future reviews of this type. In addition to presentation of data, it would help if the field could describe the behavioural elements of interventions more clearly, for example by using the CALO-RE taxonomy. Similarly, there is no current consensus on how the energy prescription is described, for example in terms of the daily deficit of energy intake or the intended weight loss; a standard approach would also help here. Finally, a suggested data set of cost, interim measures of diet and physical activity, and biomedical and anthropometric outcomes, along with recommendations as to the ways the data might be presented, including accounting for loss to follow-up, would be very welcome, and enable future synthesis to evaluate these outcomes in more depth.

In summary, most but not all behavioural weight loss programmes are effective, with the more effective programmes leading to 8 kg of weight loss in 12 months. There was strong evidence that, for programmes with a given length, more sessions led to no greater weight loss. There was evidence, potentially confounded, that asking participants to count calories led to a 3 kg greater weight loss than otherwise similar programmes that did not do this. Likewise, providing contact between participants and a dietitian was associated with a modest but worthwhile improvement of 1.5 kg greater weight loss, as was the use of behaviour change techniques that involved comparing a participant's behaviour with that of others. These findings warrant further consideration in randomized trials. However, the apparent similarity in the descriptions of programmes that varied greatly in their effectiveness hampered our ability to detect the key aspects of the interventions that led to greater effectiveness. Assessments of the delivery of programmes that differ in effectiveness are likely to prove crucial in understanding why some programmes are greatly effective, others modestly so, and others completely ineffective.

## Conflict of interest statement

The National Institute for Health and Care Excellence (NICE) provided support for the original review upon which the submitted work was based. The MRC received grants from Weight Watchers International for work where Susan Jebb was principal investigator. She received no personal remuneration in regard to this work. Susan Jebb has received personal fees from Rosemary Conley and hospitality from Weight Watchers International outside of the submitted work. Paul Aveyard has received hospitality from Weight Watchers and Slimming World outside of the submitted work. Paul Aveyard and Susan Jebb were each authors on one study included in the review.

## Abbreviations

BMI: body mass index
BWMP: behavioural weight management programme.

## Supporting Information

File S1 Review protocol. **File S2.** Medline search strategy. **Figure S1.** PRISMA flow diagram of review process. **Table S1.** Characteristics of included studies. **Table S2.** Risk of bias in included studies.
