# Supplementary material for: Effect of behavioural techniques and delivery mode on effectiveness of weight management: systematic review, meta-analysis and meta-regression
Source: Obes Rev. 2014 Mar 18;15(7):598–609. doi: 10.1111/obr.12165 (PMC4237119; doi:10.1111/obr.12165)
Supplement: File S1 — Review protocol. File S2. Medline search strategy. Figure S1. PRISMA flow diagram of review process. Table S1. Characteristics of included studies. Table S2. Risk of bias in included studies. [file obr0015-0598-SD1.pdf]

Supplemental information

Effect of behavioural techniques and delivery mode on effectiveness of weight management: systematic review, meta-analysis, and meta-regression

Jamie Hartmann-Boyce[1], David J Johns[2], Susan A Jebb[1,2], and Paul Aveyard[1], on behalf of the Behavioural Weight Management Review Group

Affiliations:
1 Department of Primary Care Health Sciences, Radcliffe Observatory Quarter, University of Oxford, Oxford, UK OX2 6GG
2 MRC Human Nutrition Research, Elsie Widdowson Laboratory, 120 Fulbourn Rd, Cambridge, UK, CB1 9NL

Corresponding author: Jamie Hartmann-Boyce, Jamie.hartmann-boyce@phc.ox.ac.uk, tel 01865 289 206, Department of Primary Care Health Sciences, Radcliffe Observatory Quarter, University of Oxford, Oxford, UK OX2 6GG

File S1. Review protocol

Note: This publication is part of a larger review and hence uses a subset of studies and methods from those described in the agreed protocol (namely, randomized controlled trials only, and methods relating to weight change and programme characteristics). Otherwise, methods are as reported in the protocol (which consisted of two parts, the 'update review' and the 'evidence review)'.

Protocol for update review: Managing overweight and obese adults: update review

NICE Reference	CPHE-URWMS-EV03-2012
Long title	The clinical effectiveness of long-term weight management schemes for adults: a systematic review
Project lead	Paul Aveyard (paul.aveyard@phc.ox.ac.uk)
Project manager	Jamie Hartmann-Boyce (Jamie.hartmann-boyce@phc.ox.ac.uk)
CPHE Technical Lead	Adrienne Cullum
CPHE Associate Director	Jane Huntley

Review team

This project will be conducted by a team of researchers from different institutions. The team members, and their roles on the review, will be:

Paul Aveyard, Professor of Behavioural Medicine, Department of Primary Care Health Sciences, University of Oxford	Lead systematic reviewer. Making key methodological choices within the systematic review. Chair meetings of the review team. Overall responsibility for delivery to NICE, ensuring report meets agreed protocol, discussing and agreeing with NICE any divergences from protocol. Writing and editing drafts and final report. Acting as third reviewer in cases of controversy.
Jamie Hartmann-Boyce, Research Associate, Department of Primary Care Health Sciences, University of Oxford	Systematic reviewer. Project managing the delivery of the various parts of the project. Working with NICE on search methods. Screening, appraisal and data extraction of included studies. Writing and editing drafts and final report.
David Johns, Investigator Scientist, MRC Human Nutrition Research	Systematic reviewer. Screening, appraisal and data extraction of included studies. Writing and editing drafts and final report.
Rafael Perera, Director Statistics Group, Department of Primary Health Care Sciences, University of	Statistics advice.

Oxford	
Igho Onakpoya, Researcher in Pharmacovigilance, Department of Primary Health Care Sciences, University of Oxford	Systematic reviewer. Assisting with data extraction.

Note: The search will be run by Daniel Tuvey at NICE, with input from Jamie Hartmann-Boyce.

Advisory team

In addition to the core project team, we have a team of advisors who the core team will call upon the on matters relating directly to their areas of expertise, as identified below.

Carolyn Summerbell, Professor of Human Nutrition and Principal of John Snow College, Durham University	Advice on matters relating to systematic review methodology
Jane Ogden, Professor in Health Psychology, Department of Psychology, University of Surrey	Guidance on psychological theories and patients views and perceptions regarding weight loss programmes
Susan Jebb, Head of Department, Diet and Population Health, MRC Human Nutrition Research	Advice in relation to dietary prescriptions
Dawn Phillips, Public Health Portfolio Lead for Adult Obesity and Physical Activity, County Durham	Guidance on clinical aspects
Igho Onakpoya, Researcher in Pharmacovigilance, Department of Primary Care Health Sciences, University of Oxford	Advice on systematic review methodology

Key deliverables and dates

**Deliverable**	**Date**	**Comments back from NICE CPHE by:**
1st Draft review protocol	19 October 2012	26 October 2012
Revised review protocol	30 October 2012	2 November 2012
Signing-off of review protocol	7 November 2012	
Signing-off of search strategy	5 November 2012	
Interim progress meeting/ teleconference (1) –	21 November	
Interim progress meeting/ teleconference (2) –	19 December 2012	
Draft report submitted to NICE	18 January 2013	25 January 2013
Amended report submitted to NICE	11 February 2013	

Slides for PDG meeting submitted to NICE	19 February 2013	
Review presented to PDG	26 February 2013	
Final review submitted	13 March 2013	

Context

This Review Protocol is for Review 1, with the first draft submitted by the agreed delivery date of 18 January 2013, and the final review to be submitted by 13 March 2013. A separate but related evidence review (Review 2) is covered in a separate protocol. As this is an update of an existing review (Loveman et al 2011[1]), the scope is unlikely to change beyond what is agreed here.

Purpose of this document

This document describes the aims, scope and intended methods of the update review which will be produced to support the development of NICE Public Health Guidance on lifestyle weight management programmes for overweight and obese adults.

Unless otherwise stated in this Review Protocol, this review, and its report will be conducted according to the rigorous methods described in the Cochrane Handbook, the York Centre for Reviews and Dissemination Handbook, and the 2nd Edition of the *Methods for the development of NICE public health guidance* (2009). As this is an update review it will follow as closely as possible the scope and format of the original review (Loveman 2011) to enable direct comparison between the two, and the use of the two reviews in conjunction with one another. Where there is a discrepancy between Loveman's reporting methods and those suggested by the above listed handbooks, CPHE will be consulted.

Clarification of scope

This review aims to inform readers about the relative importance of the components included in multi-component lifestyle interventions for the treatment of obesity. This review will therefore cover only those interventions that include both a diet and exercise component, and will exclude referral to individual clinicians, management of associated conditions, surgery, and pharmacological treatments. The review will be restricted to interventions that are judged to be feasible for implementation in the UK.

For the remainder of the document, multi-component lifestyle weight management programs (LWMPs) will be defined as those which focus on reducing energy intake, increasing physical activity and changing behaviour. These may include weight management programmes, courses or clubs:

- specifically designed for adults who are obese or overweight
- that accept adults through self-referral or referral from a health practitioner
- provided by the public, private or voluntary sector
- based in the community, workplaces, primary care or online.

[1] Loveman E, Frampton GK, Shepher J, Picot J, Cooper K, Bryant J, et al. The clinical effectiveness and cost-effectiveness of long-term weight management schemes for adults: a systematic review. *Health Technology Assessment* 2011;15(2).

Review questions

The primary question in this review is similar to that of Loveman 2011, though this update will not focus on cost-effectiveness. The primary question is therefore:

- How effective and cost-effective are multi-component lifestyle weight management programmes for adults?

We will also attempt to answer secondary questions relating to these programmes. Should data be available, we will attempt to answer:

- How does effectiveness vary for different population groups (for example, men, black and minority ethnic or low-income groups)?
- How does effectiveness and cost effectiveness vary based on the components of the individual programmes (including behavioural or psychological components)?
- Are there any adverse or unintended effects associated with the use of LWMPs?

Factors which influence the effectiveness, implementation or sustainability of initiatives may be either positive ('facilitators') or negative ('barriers'), and will also be explored when assessing the included studies. However, detailed questions about key components of LWMPs, their implementation, user experience, and facilitators and barriers (overall and for specific population groups) will be addressed separately in review 2. Review 1 will focus only on the effectiveness of the LWMPs.

Outcomes

We will extract and report data on the following outcomes:

- Quantitative changes in anthropometric measures – weight, BMI, waist circumference, etc
- Intermediate measures of diet and physical activity
- Process measures such as participant satisfaction with weight management services, adherence to the intervention and attendance at sessions
- Economic outcomes (narrative only)
- Adverse effects

Inclusion criteria

For the clinical effectiveness review, we propose to follow similar criteria for including and excluding studies as used in the Loveman 2011 report, with two key changes: we will not include LWMPs that involve medications for obesity of any type, unless their use is not part of the LWMP and is comparable in both intervention and control groups; and we will include studies with 12 month follow-up or longer (Loveman required a minimum of 18 months follow-up, we will examine those studies excluded from Loveman on the basis of too short a follow-up period.. The revised inclusion criteria are listed below.

Population

- Adults (≥ 18 years) classified as overweight or obese, i.e. people with a BMI of ≥ 25 kg/m2 and ≥ 30 kg/m2, respectively.
- Studies in children, pregnant women, and people with eating disorders were not included, nor were studies specifically in people with a pre-existing medical condition such as diabetes, heart failure, uncontrolled hypertension or angina.

Intervention

- Structured, sustained multi-component weight management programmes (i.e. the intervention had to be a combination of diet and physical activity with a behaviour change strategy to influence lifestyle).
- Components of the programme had to be clearly specified (i.e. details provided of the diet, behavioural definition, and exercise components; see below).
- Programmes that included a long-term follow-up of more than 12 months.
- The programme was delivered by the health sector, in the community or commercially.
- Multi-component programmes that involved the use of any surgery or medication, over-the-counter or otherwise, are excluded.
- Interventions incorporating other lifestyle changes such as efforts at smoking cessation or reduction of alcohol intake were not included.

Comparators

- Normal practice (as defined by the study).
- Single-component weight management strategies.
- Other structured multi-component weight management programmes.

Outcomes

- Studies were required to include a measure of weight loss.

Types of studies

- RCTs only.
- Studies published as abstracts or conference presentations were only included if sufficient details were presented to allow an appraisal of the methodology and the assessment of results to be undertaken.
- Case series, case studies, cohort studies, narrative reviews, feasibility studies, editorials and opinions were not included.
- Systematic reviews were used as a source of references.

Location

- Undertaken in any setting (i.e. community, commercial, primary care, online).
- Studies conducted in OECD countries will be considered for inclusion.[2] In the instance that a study has been conducted in an OECD country but the reviewers and advisory panel judge that

---

[2] The original scope specified studies in the UK only. The extension to OECD countries has been agreed with NICE with the understanding that the completion of the review by stated dates is the key priority, and that the revised scope can be limited to UK only countries if the schedule so requires.

the intervention would not be feasible for implementation in the UK, the reviewers will consult with CPHE regarding its inclusion.

- Studies conducted in non OECD countries will be excluded.

Cost effectiveness

As per Loveman 2011, references identified by the search strategy for the systematic review of cost-effectiveness will be considered for inclusion only if:

- They report both health service costs and effectiveness of multicomponent adult weight management programmes

OR

- Present a systematic review of such evaluations

Unlike Loveman, initially, only UK cost effectiveness studies will be included in the search, but if this results in too few studies being included, we will consult NICE to agree on a wider search being undertaken (likely all English language OECD countries).

Specification of components of intervention

Loveman et al required that, in order for a study to be included, at least two items under each of the below components (diet, exercise, and behaviour modification) had to be specified.

Diet

- type of diet
- calories
- proportion of diet (e.g. proportion of diet made up of fats, protein, carbohydrate)
- monitoring

Exercise

- mode
- type
- frequency/length sessions
- delivered by
- level of supervision
- monitoring

Behaviour modification

- mode
- type
- content
- frequency/length sessions
- delivered by.

Where studies are multicomponent but the study report does not meet the above criteria, we will follow the below approach:

- If the study identifies that the intervention is a defined weight loss programme (commercial or otherwise), we will search online for details of the weight loss programme and use these to classify the study components. Where insufficient details are available online, we will contact the programme directly, specifying that a response will be needed by 10 December 2012.

- If the study is not of an identifiable and defined weight loss programme, we will email study authors with a template email asking them to provide any details they have on the above elements, specifying that a response will be needed by 10 December 2012.
- Where authors do not respond by the deadline specified, provide insufficient information, or where we cannot find a current e-mail address, the study will be excluded, with the reason for exclusion clearly identified (for example, "unclear detail on physical activity component").

Search methods

This is an update of an existing review and as such the existing search strategy as published in Loveman 2011 will be used. The literature search will be run by NICE with input from one reviewer (Jamie Hartmann-Boyce). Searches will be fully documented and references will be stored in a Reference Manager database.

The detailed search strategy will be agreed separately between reviewers and the CPHE's information specialist (see schedule). Any adaptations to the Loveman 2011 strategy will be confirmed with NICE and are likely to be related to increasing the specificity of the search, given the time constraints involved.

Study selection at search stage

- Studies indexed since date of last Loveman search (December 2009)
- Studies conducted in OECD countries.

In addition to running the updated searches specified above, we are aware that Loveman has excluded some diabetes prevention studies which meet the above inclusion criteria (ie lifestyle interventions for overweight and obese adults, pre-existing clinical condition not a prerequisite for study enrollment). After discussion with NICE, we have agreed to include these studies. These have not been explicitly excluded from Loveman so there is no means of gathering a quick list of these studies. Instead, to ensure we have not missed major trials in this area published prior to the period of our updated search, we will use published reviews of diabetes prevention trials to identify relevant studies.

Study selection process

Assessment for inclusion will be undertaken initially at title and/or abstract level (to identify potential papers/reports for inclusion) by a single reviewer (and a sample checked by a second reviewer), and then by examination of full papers. A third reviewer will be used to help adjudicate inclusion decisions in cases of disagreement. Where the research methods used or type of initiative evaluated are not clear from the abstract, assessment will be based upon a reading of the full paper.

Quality assessment and data extraction

For the review of clinical effectiveness, we will critically appraise the literature for inclusion using a checklist based on the York CRD approach and as described in the CPHE manual.[18] However, we will modify this slightly for behavioural intervention trials and will not evaluate included studies on the

basis of blinding. We will present the appraisal in tables and summarise the findings in text as described in the CPHE manual.

Data extraction will be conducted using a pre-specified data extraction form, which will be piloted by two reviewers before its use. Data extraction and quality assessment will be done independently by two reviewers, who will then compare data extraction forms. Any discrepancies will be resolved by discussion or, where needed, by referral to a third reviewer.

If deemed to be helpful for the write-up, we will reference data extracted as part of the Loveman 2011 review, but in narrative elements of the write-up we will use the data extracted by the Loveman et al rather than re-extracting these data ourselves (full, completed data extraction forms are published in the appendices of Loveman). If we conduct meta-analyses or meta-regression (see next section), we will re-extract key outcomes from the included studies in Loveman to ensure we are using the same approach to data across all studies included in the analysis.

For the review of cost-effectiveness, we will critically appraise the literature using Lovemans' *Critical appraisal checklist of economic evaluation* (table 23, page 53). Elements of this table refer to applicability to the UK; if as discussed above we do not include cost-effectiveness literature from outside the UK, we will remove these items from the checklist. All other items will remain the same.

Data synthesis and presentation, including evidence statements

We will synthesise the data in narrative form, as Loveman et al did. However, we will consider whether meta-analysis and meta-regression could be undertaken and use the baseline observation carried forward approach with standard errors calculated as described recently.[3] This is likely to be an exploratory technique rather than a definitive guide to a single underlying effect size, and such analyses will only be conducted if appropriate data is available and if time allows.

If data and time allow, we will run a meta-regression on variables of LWMPs. Meta-regression will allow us to explore whether outcomes are associated with the various characteristics of the interventions and this will prove especially useful when it comes to giving guidance on Review 2 questions. Regardless of whether a meta-regression is performed, we will categorise studies based on the following elements (taken from Jolly et al[4]):

- Professional background of therapies
- Training of therapist
- Assessment of therapist's competence
- Fidelity checking of intervention
- Group or individual
- Duration of sessions, frequency, programme length and setting
- Content of sessions
- Weight loss goal
- Relative emphasis on diet and exercise

[3] Kaiser KA, Affuso O, Beasley TM, Allison DB. Getting carried away: a note showing baseline observation carried forward (BOCF) results can be calculated from published complete-cases results. Int J Obes 2012; 36(6):886-889.

[4] Jolly K, Lewis A, Beach J, Denley J, Adab P, Deeks JJ et al. Comparison of range of commercial or primary care led weight reduction programmes with minimal intervention control for weight loss in obesity: Lighten Up randomised controlled trial. BMJ 2011; 343.

- Intervention theoretical background
- Predominant behavioural change techniques used

Behavioural change techniques will be assessed through the use of a pre-defined taxonomy, included as an element of the data extraction process. Each included study will be assessed against a checklist of the taxonomy, with a dichotomous yes/no option for the reviewer to indicate if the intervention included that behavioural element. The description will be obtained through the study report, and hence it should be noted that the application of the taxonomy will be limited by the depth of description provided in the report. We will use the 40-item refined taxonomy of behaviour change techniques to help people change their physical activity and healthy eating behaviours (the CALORE taxonomy) as defined by Michie et al.[5]

Where possible, we will draw weight curves for each study, mapping weight change during intervention and weight change after intervention end and seek to summarise these as appropriate.

We will group studies by the nature of the comparison, including the nature of the control group. We will note whether the control group received an active treatment that might be expected to lower weight gain or not and try to account for this in the analysis. We will also describe the nature of the intervention e.g. the energy prescription/deficit given, the intensity of the physical activity prescription, the length of the programme, and any ongoing support offered. If possible, we will calculate the energy expenditure prescription in METs so that it will be possible to compare energy restriction with increased energy burning.

Data synthesis and presentation, including evidence statements, will be conducted according to the procedures outlined in the 2nd Edition of *Methods for development of NICE public health guidance 2009* where appropriate.

Key choices in how to synthesise the included evidence, or in how to develop evidence statements for this review, will be discussed with the relevant analysts at CPHE.

---

[5] Susan Michie, Stefanie Ashford, Falko F. Sniehotta, Stephan U. Dombrowski, Alex Bishop & David P. French (2011): A refined taxonomy of behaviour change techniques to help people change their physical activity and healthy eating behaviours: The CALO-RE taxonomy,
Psychology & Health, 26:11, 1479-1498

Protocol for Evidence Review: managing overweight and obese adults, evidence review

NICE Reference	CPHE-URWMS-EV03-2012
Long title	The clinical effectiveness of long-term weight management schemes for adults: a systematic review
Project lead	Paul Aveyard (paul.aveyard@phc.ox.ac.uk)
Project manager	Jamie Hartmann-Boyce (Jamie.hartmann-boyce@phc.ox.ac.uk)
CPHE Technical Lead	Adrienne Cullum
CPHE Associate Director	Jane Huntley

Review team

This project will be conducted by a team of researchers from two different institutions. The team members, and their roles on the review, will be:

Paul Aveyard, Professor of Behavioural Medicine, Department of Primary Care Health Sciences, University of Oxford	Lead systematic reviewer. Making key methodological choices within the systematic review. Chair meetings of the review team. Overall responsibility for delivery to NICE, ensuring report meets agreed protocol, discussing and agreeing with NICE any divergences from protocol. Writing and editing drafts and final report. Acting as third reviewer in cases of controversy.
Jamie Hartmann-Boyce, Research Associate, Department of Primary Care Health Sciences, University of Oxford	Systematic reviewer. Project managing the delivery of the various parts of the project. Working with NICE on search methods. Screening, appraisal and data extraction of included studies. Writing and editing drafts and final report.
David Johns, Investigator Scientist, MRC Human Nutrition Research	Systematic reviewer. Screening, appraisal and data extraction of included studies. Writing and editing drafts and final report.
Rafael Perera, Director Statistics Group, Department of Primary Health Care Sciences, University of Oxford	Statistics advice.

Advisory team

In addition to the core project team, we have a team of advisors who the core team will call upon for matters relating directly to their areas of expertise, as identified below.

Carolyn Summerbell, Professor of Human Nutrition and Principal of John Snow College, Durham	Advice on matters relating to

University	systematic review methodology
Jane Ogden, Professor in Health Psychology, Department of Psychology, University of Surrey	Guidance on psychological theories and patients views and perceptions regarding weight loss programmes
Susan Jebb, Head of Diet and Population Health, MRC Human Nutrition Research	Advice in relation to dietary prescriptions and weight management
Dawn Phillips, Public Health Portfolio Lead for Adult Obesity and Physical Activity, County Durham	Guidance on clinical aspects
Amanda Lewis, NIHR SPCR Research Fellow, Department of Primary Care Health Sciences, University of Oxford	Guidance on research into weight management in primary care
Igho Onakpoya, Researcher in Pharmacovigilance, Department of Primary Care Health Sciences, University of Oxford	Systematic reviewer. Data extraction of included studies.

Key deliverables and dates

**Deliverable**	**Date**	**Comments back from NICE CPHE by:**
1st Draft review protocol	15/2/13	
Revised review protocol	25/2/13	25/2/13
Signing-off of review protocol	27/2/13	
Signing-off of search strategy	n/a	
Interim progress teleconference–	6th March<br>20th March<br>4th April	
Draft report submitted to NICE ("drip feeding approach" as per Review 1a)	7 March 2013 – 21 March	14 March (on components submitted 7 March)
Amended report submitted to NICE	28 March	
Slides for PDG meeting submitted to NICE	11 April	
Review presented to PDG	16 April	
Final review submitted	30 April	

Context

This Review Protocol is for Review 1b. Review 1a, which will be presented in final form on 11.2.13 in response to fulfilment of the tender for the Update Review, commissioned by NICE. There were substantial overlaps between the two reviews. In agreement with NICE, we agreed to defer some analyses for a separate review, this is Review 1b, which also incorporates some questions from the Evidence Review tender.

Purpose of this document

This document describes the aims, scope and methods of Review 1b, which will be produced to support the development of NICE Public Health Guidance on lifestyle weight management programmes for overweight and obese adults.

Unless otherwise stated in this Review Protocol, this review, and its report will be conducted according to the rigorous methods described in the Cochrane Handbook, the York Centre for Reviews and Dissemination Handbook, and the 2nd Edition of the *Methods for the development of NICE public health guidance* (2009).

Clarification of scope

The aim of this review is to examine

1. How components of behavioural weight loss programmes affect the outcome. (This is question 2 of the Evidence Review tender)
2. What happens to the difference in weight between people treated on a behavioural weight loss programme and a control group in the longer term (once the intervention has ended)? How quickly does weight increase after the end of the programme and do the characteristics of the programme affect the rate of increase in weight? (These questions are not specified in the tender but the review team think that they are important and useful).

3. What interventions can maintain weight loss after the end of a behavioural weight loss programme? (This is question 4 of the Evidence Review tender).
4. Is there evidence to support the best practice principles that NICE proposed in its 2006 guidance? (This is question 1 of the Evidence Review).

How components of behavioural weight loss programmes affect the outcome

This is phrased in the tender as "What are the most effective and cost effective behavioural or psychological components of a lifestyle weight management programme for adults – and who might best deliver them?"

The data to answer this question will come from Review 1a and a review of a further group of trials that were uncovered during the search for studies for Review 1a. The trials in Review 1a were defined as behavioural weight loss programmes that incorporated dietary and physical activity interventions versus a control group. The control interventions were rarely no intervention at all, but we included the following as unlikely to be providing much active treatment

1. No intervention at all or leaflet/s only[6]
2. Discussion/advice/counselling in one-off session +/-leaflet
3. Seeing someone more than once for discussion of something other than weight loss.
4. Seeing someone more than once for weight management, person untrained +/- leaflets

A fifth group of studies includes those that have a behavioural weight management programme that incorporates only physical activity or diet but not both, and a sixth group of studies includes behavioural programmes with both diet and physical activity components. In this review, we will appraise such papers as were found and catalogued in Review 1a and incorporate those arms of trials excluded from Review 1a that have interventions of this type.

In Review 1a we reviewed the effectiveness of 44 different interventions and we split the interventions versus control comparisons using subgroup analyses. We considered the following questions:

1. Whether the programme is delivered in groups or individually
2. The length of the programme
3. Whether the aim was weight loss or diabetes prevention
4. Whether the programme was delivered remotely, for example by Internet, or face-to-face
5. Supervised versus recommended exercise programme
6. Energy prescription target or no target
7. Frequency of contact with participants

In addition, in Review 1b, we will consider an eighth question

---

[6] Note that leaflets included static websites, i.e. information and advice only, not interactive weight loss programmes, which come under 5 or 6).

8. Are the behavioural change techniques used associated with improved effectiveness

The one element that requires explanation in this list is the behavioural change techniques. These are elements of the behavioural programme that can be used to encourage behaviour change. At the simplest, this can include advice giving. The taxonomy has been developed to allow researchers to describe behavioural counselling in standardised ways that allow comparison across studies.(Abraham & Michie 2008;Michie et al. 2011)

As described in Review 1a, we extracted data on the behaviour change techniques (BCTs) used to try to motivate and support individuals to change their behaviour. We said "Behavioural change techniques will be assessed through the use of a pre-defined taxonomy, included as an element of the data extraction process. Each study will be assessed against a checklist of the taxonomy, with a yes/unclear/no option for the reviewer to indicate if the intervention included that behavioural element. The description will be obtained through the study report, and hence it should be noted that the application of the taxonomy will be limited by the depth of description provided in the report. We will use the 40-item refined taxonomy of behaviour change techniques to help people change their physical activity and healthy eating behaviours (the CALORE taxonomy) as defined by Michie et al.[7]" Items were coded as U where the technique was not explicitly stated but reviewers agreed it was implied. Michie and colleagues have grouped these 40 BCTs together using a grouping system (Table 1), which is essential for meaningful meta-analysis or meta-regression. We will give each BCT within each category a score: 0 if it is not used, 0.5 if the description was unclear, and 1 if the technique is clearly used. We will total these within categories as a measure of the emphasis of a particular intervention on BCTs of that type. One item on the CALORE taxonomy (27 – use of follow-up prompts) was not assigned to a BCT category and will be assessed independently.

[7] Susan Michie, Stefanie Ashford, Falko F. Sniehotta, Stephan U. Dombrowski, Alex Bishop & David P. French (2011): A refined taxonomy of behaviour change techniques to help people change their physical activity and healthy eating behaviours: The CALO-RE taxonomy,
Psychology & Health, 26:11, 1479-1498

Table 1 BCTs from the CALORE taxonomy grouped as proposed by Michie and colleagues

Technique group	Taxonomy item
Goals and planning	05- Goal setting (behaviour)<br>06- Goal setting (outcome)<br>07- Action planning<br>08- Barrier identification/problem solving<br>10- Prompt review of behavioural goals<br>11- Prompt review of outcome goals<br>20- Provide information on where and when to perform the behaviour<br>25- Agree behavioural contract<br>35- Relapse prevention/coping planning
Reward and threat	12- Prompt rewards contingent on effort or progress towards behaviour<br>13- Provide rewards contingent on successful behaviour<br>14- Shaping<br>32- Fear arousal<br>40- Stimulate anticipation of future rewards
Regulation	36- Stress management/emotional control training<br>38- Time management
Antecedents	24- Environmental restructuring
Identity	30- Prompt identification as role model/position advocate
Self-belief	18- Prompting focus on past success<br>33- Prompt self talk
Covert learning	34- Prompt use of imagery
Feedback and monitoring	16- Prompt self-monitoring of behaviour<br>17- Prompt self-monitoring of behavioural outcome<br>19- Provide feedback on performance
Social support	29- Plan social support/social change<br>37- Motivational interviewing<br>39- General communication skills training
Shaping knowledge	21- Provide instruction on how to perform the behaviour
Natural consequences	01- Provide information on consequences of behaviour in general<br>02- Provide information on consequences of behaviour to the individual<br>31- Prompt anticipated regret
Comparison of behaviour	03- Provide information about others' approval<br>04- Provide normative information about others' behaviour<br>22- Model/Demonstrate the behaviour<br>28- Facilitate social comparison
Associations	23- Teach to use prompts/cues
Repetition and substitution	09- Set graded tasks<br>15- Prompting generalisation of a target behaviour<br>26- Prompt practice

Whereas in Review 1a we used subgroup analysis to investigate differences in effectiveness, in Review 1b we will use meta-regression. Meta-regression is more powerful because it affords us the ability to examine the effects of interventions characterised in one way while accounting for other differences between programmes. However, with 40 intervention-control comparisons, it is possible to include a maximum of four predictors to avoid over-fitting the model. Therefore there is limited scope to address all differences between

programmes. Where data exist, we will use within trial data to examine some of these questions and use the totality of evidence to draw conclusions.

What happens to the difference in weight between people treated on a behavioural weight loss programme and a control group in the longer term?

This questions relates to the maintenance of weight loss achieved by behavioural weight loss programmes. The review team will report data from Review 1a that includes:

- A trajectory of weight change for all studies.
- A meta-regression to examine whether the weight trajectory after programme end depends upon the characteristics discussed above ('How components of behavioural weight loss programmes affect the outcome'). For this analysis, we will ignore the initial weight loss and will look at how weight changes that occur after the end of the programme vary among the programme types.
- A meta-analysis where possible of within study data of trials that randomised participants to longer or shorter behavioural weight loss programmes
- A meta-regression of between study data of trials that compared behavioural weight loss programmes to control and where the length of the programme varied between studies

What interventions can maintain weight loss after the end of a behavioural weight loss programme?

To answer this question we will conduct a review of reviews with the below inclusion criteria.

Inclusion criteria

Population

- Adults (≥ 18 years) initially classified as overweight or obese prior to starting a weight loss programme, i.e. people with a BMI of ≥ 25 kg/m2 and ≥ 30 kg/m2, respectively. Enrolment in a weight loss maintenance intervention implies that people who have lost weight are enrolled. We propose no restrictions on how much weight loss has been achieved prior to enrolment in a weight loss maintenance trial.
- Reviews of trials in children, pregnant women, and people with eating disorders will not be included, nor studies specifically in people with a pre-existing medical condition such as diabetes, heart failure, uncontrolled hypertension or angina.

Intervention

Any intervention aimed at maintenance of weight loss that is not pharmacotherapy or surgery

Control

Usual care or other control condition

Types of studies

A weight loss maintenance study enrols participants who have already lost weight by means other than surgery.

Reviews of randomised controlled trials, whether systematic or unsystematic, will be included. We will not include reviews of observational studies that compare the characteristics of weight loss maintainers to those who regain weight.

Location

- Undertaken in any setting
- Studies in any country will be included, though we anticipate that reviews are likely to include overwhelmingly studies conducted in OECD countries.

Search methods

The aim is to be systematic but not comprehensive and thus the searches will concentrate on specificity over sensitivity. We have already established that there are no specific MeSH terms for weight loss maintenance. Therefore our search strategy for Review 1a, which included systematic reviews, will have located such reviews. We will therefore rerun our searches for Review 1a but remove the date restriction. We will use text word searches for relevant terms, such as 'maintenance' and 'review', to find reviews of weight loss maintenance in the thousands of papers retrieved during the search for Review 1a. In addition, we will include other reviews on the topic that are referenced in the reviews that we find as a result of this search.

Study selection process

Assessment for inclusion will be undertaken initially at title and/or abstract level (to identify potential reviews for inclusion) by a single reviewer and then by examination of full papers. A second reviewer will be used to help adjudicate inclusion decisions. Where the abstract is unclear, assessment will be based upon a reading of the full paper.

Quality assessment

One reviewer will appraise reviews using the methods for appraisal of reviews described in CPHE manual. We will produce a table relating to each review and assess its quality.

Data synthesis and presentation, including evidence statements

We will extract data on the strength of evidence for particular interventions in each review and also the applicability of the evidence to the target population. We will synthesise this narratively across reviews to examine a range of interventions that affect weight loss maintenance. It is important to note that this review will exclude behavioural weight loss programmes unless such programmes have enrolled participants who have already lost weight. Randomised trials of longer versus shorter weight loss programmes are included in Review 1a.

Is there evidence to support the best practice principles that NICE proposed in its 2006 guidance?

The current best practice principles are taken from existing NICE guidance on obesity, CG43:

The best practice principles identified in NICE guidance on management of obesity are:

Primary care organisations and local authorities should recommend to patients, or consider endorsing, self-help, commercial and community weight management programmes only if they follow best practice [4] by:

- helping people assess their weight and decide on a realistic healthy target weight (people should usually aim to lose 5–10% of their original weight)
- aiming for a maximum weekly weight loss of 0.5–1 kg
- focusing on long-term lifestyle changes rather than a short-term, quick-fix approach
- being multicomponent, addressing both diet and activity, and offering a variety of approaches
- using a balanced, healthy-eating approach
- recommending regular physical activity (particularly activities that can be part of daily life, such as brisk walking and gardening) and offering practical, safe advice about being more active
- including some behaviour change techniques, such as keeping a diary and advice on how to cope with 'lapses' and 'high-risk' situations
- recommending and/or providing ongoing support.

The data to address the question of whether these principles are evidence based will be derived from the data in Review 1a, for which there is a detailed protocol. If there are no data available in the review that are relevant, we will perform a bespoke search and, depending on the data available, may also refer to other published guidelines.

Principles: helping people assess their weight and decide on a realistic healthy target weight (people should usually aim to lose 5–10% of their original weight) and aiming for a maximum weekly weight loss of 0.5–1 kg/week

For each study in Review 1a we extract whether or not a target was set and what that target was. We will use meta-regression to examine whether studies that set targets and the weight loss target is associated with greater weight loss. However, there are several caveats. First, the nature of behavioural weight loss programmes under study is that they tend not to have very extreme goals so that there may be little variation between studies. Second, there are many dimensions on which programmes might vary and it is impossible statistically to control for all such variations and many variations will not be recorded.

The main programmes that do aim for rapid weight loss are very low calorie diets (VLCDs). However, the effectiveness of setting high weight loss goals in VLCD programmes is confounded with providing meals, which is a universal feature of VLCDs. Meal replacement was a feature of only a few of the included studies in Review 1a, so assessing the effectiveness of extreme weight loss goals net of the effect of meal replacement is challenging as there are too few behavioural weight management interventions that aimed for moderate weight loss and yet which provided meals, in the way that VLCD programmes do.

We found two programmes that incorporated VLCDs in Review 1a. These were Wadden (1988), which includes very few participants, and Weinstock (1998), which also includes few participants and has no usable outcome data presented in the paper. However, for work outside the NICE review, we have systematically searched for reviews of VLCDs, which yielded a recent systematic review (Mulholland 2012). We will examine the reviews to assess whether there is evidence that the rapid weight loss typically induced by VLCDs results in weight regain. This will be a narrative synthesis .

Principle: focusing on long-term lifestyle changes rather than a short-term, quick-fix approach

We will use data from Review 1a, considering those studies that compare lifestyle weight management programmes with a diet only comparator that lasts for less than 6 months. A 6 month cut off was chosen because subgroup analysis from Review 1a suggested that studies less than 6 months were not as effective as those last 6+ months.

Principle: being multicomponent, addressing both diet and activity, and offering a variety of approaches

Review 1a examines the effectiveness of multicomponent lifestyle programmes compared with no intervention. As outlined above, in Review 1b, we will examine trials of the effectiveness of diet and physical activity interventions compared with diet only and physical activity only weight loss programmes. Meta-analysis will be used to compare programmes that include both physical activity and dietary behaviour change to programmes that include only one of those elements.

Principle: using a balanced, healthy-eating approach

We will use data from Review 1a, looking specifically at studies which compare BWMPs with comparator arms where no dietary advice has been given.

Principle: recommending regular physical activity (particularly activities that can be part of daily life, such as brisk walking and gardening) and offering practical, safe advice about being more active

In Review 1b we will characterise interventions by the type of physical activity that they promote. We will classify the activities in the programme as easy to incorporate or specific exercise activities and use meta-regression to examine whether there is evidence that programmes that include this kind of activity are more effective than programmes that include other forms of activity.

Principle: including some behaviour change techniques, such as keeping a diary and advice on how to cope with 'lapses' and 'high-risk' situations

By definition, all multicomponent behavioural weight management programmes include behavioural change techniques. The key question is which techniques are associated with greater effectiveness. We are investigating these as described above.

Principle: recommending and/or providing ongoing support.

The contrast with offering ongoing support is to offer one-off advice on how to lose weight. In Review 1a we investigated whether programmes in which participants were randomised to advice, usually a single session of advice by an untrained advisor, or to a programme of ongoing support. There was convincing evidence that programmes with ongoing support were more effective than one or two episodes of advice.

In addition, the trials in Review 1a randomised participants to BWMP or control, but the BWMPs varied in length trials of programmes compared long programmes to control, while others compared short programmes to control. We will use meta-regression on the studies in Review 1b to examine whether there is data that support the notion that longer support is more effective than shorter support. We will also use meta-analysis and meta-regression to compare the effectiveness of programmes in which contact frequency or intensity declined over time (for example, initially in

person sessions but then phone sessions, or initially weekly declining to monthly to trials where the intervention was of consistent intensity and ended abruptly. These data will be derived from taxonomy item 27 – use of follow-up prompts).

File S2. MEDLINE search strategy

Database: **Ovid MEDLINE(R)** 1946 to November Week 1 2012 (searched 07 November 2012)

Strategy used:

1	Obesity/ or Obesity, Morbid/ or Obesity, Abdominal/
2	exp weight gain/
3	Overweight/
4	(overweight or over weight or overeat* or over eat* or overfeed* or over feed*).ti,ab.
5	(weight adj1 gain*).ti,ab.
6	obes*.ti,ab.
7	or/1-6
8	(modific* or therap* or intervention* or strateg* or program* or management or scheme* or group* or pathway*).ti,ab.
9	(weight adj1 los*).ti,ab.
10	(weight adj1 reduc*).ti,ab.
11	exp weight loss/
12	8 and (9 or 10 or 11)
13	Obesity/dh, pc, th
14	Obesity, Morbid/pc, dh, th
15	8 and (13 or 14)
16	Diet Therapy/
17	Diet, Fat-Restricted/
18	Diet, Reducing/
19	Dietetics/ed, mt
20	(diet or diets or dieting).ti,ab.
21	(low calorie or hypocaloric or calorie control*).ti,ab.
22	(health* adj1 eating).ti,ab.
23	(diet* adj2 (modific* or therapy or intervention* or strateg* or program* or management or scheme*)).ti,ab.
24	(nutrition adj2 (modific* or therapy or intervention* or strateg* or program* or management or scheme*)).ti,ab.
25	(Weight Watchers or weightwatchers).ti,ab.
26	(slimming world or slimmingworld).ti,ab.
27	(lighterlife or "lighter life").ti,ab.
28	or/16-27
29	8 and 28
30	exp exercise/
31	exercise therapy/
32	(exercise and (therapy or therapies or activity or activities or class* or program* or group* or session* or scheme*)).ti,ab.
33	(Gym and (trainer* or therap* or activit* or class* or program* or group* or session* or scheme* or

	club*)).ti,ab.
34	(walk* or step* or jog* or run*).ti,ab.
35	(aerobic* or physical therap* or physical activit*).ti,ab.
36	(fitness adj (class or regime* or program* or group* or session* or scheme*)).ti,ab.
37	(reduc* adj2 sedentary behavio?r).ti,ab.
38	(dance and (therap* or activit* or class* or program* or group* or session* or scheme*)).ti,ab.
39	personal trainer*.ti,ab.
40	(gym or gyms or gymnasium*).ti,ab.
41	or/30-40
42	8 and (30 or 31 or 34 or 35)
43	32 or 33 or 36 or 37 or 38 or 39 or 40 or 42
44	cognitive therapy/
45	Counseling/
46	behavior therapy/
47	cognitive therapy/
48	behavio?ral intervention*.ti,ab.
49	(change* adj2 lifestyle*).ti,ab.
50	(changing adj2 lifestyle*).ti,ab.
51	(lifestyle adj2 modif*).ti,ab.
52	Hypnosis/
53	Counseling/
54	(counseling or counselling).ti,ab.
55	or/44-54
56	Randomised Controlled Trials as Topic/
57	randomised controlled trial.pt.
58	controlled clinical trial.pt.
59	Controlled Clinical Trial/
60	placebos/
61	random allocation/
62	Double-Blind Method/
63	Single-Blind Method/
64	(random* adj2 allocat*).tw.
65	placebo*.tw.
66	((singl* or doubl* or trebl* or tripl*) adj (blind* or mask*)).tw.
67	Research Design/
68	((random* or control*) adj5 (trial* or stud*)).tw.
69	Clinical Trials as Topic/
70	randomly.ab.
71	(randomised or randomized).ab.
72	Evaluation studies as topic/

73	comparative study/
74	(matched communities or matched populations).mp.
75	(control* adj (trial* or stud* or evaluation*)).mp.
76	(comparison group* or control* group*).mp.
77	Matched-Pair Analysis/
78	matched pair*.ti,ab.
79	Meta-Analysis/
80	meta analy*.ti,ab.
81	"Outcome Assessment (Health Care)"/
82	outcome stud*.ti,ab.
83	intervention studies/
84	follow up studies/
85	(systematic* adj (review* or methodolog* or research* or search*)).ti,ab.
86	((hand or manual or computer or electronic or database) and search*).ti,ab.
87	(hand adj search*).ti,ab.
88	(medline or embase or Cochrane or cinahl or psychlit or psychinfo or scisearch or pubmed).ab.
89	Health technology assessment*.ab,in.
90	(pooled adj analys*).ti,ab.
91	(electronic* adj search*).ti,ab.
92	(synthes* adj5 (literature* or research* or studies or data)).ti,ab.
93	or/56-92
94	12 or 15
95	7 and 93 and 94
96	7 and 28 and 93
97	7 and 29 and 93
98	7 and 41 and 93
99	7 and 43 and 93
100	7 and 55 and 93
101	96 or 98 or 100
102	97 or 99 or 100
103	96 and 98 and 100
104	96 and 98
105	96 and 100
106	98 and 100
107	104 or 105 or 106
108	97 and 99
109	97 and 100
110	99 and 100
111	108 or 109 or 110
112	103 or 107 or 111

113	Anti-Obesity Agents/
114	(sibutramine or orlistat or rimonabant).ti,ab,nm.
115	exp Bariatric Surgery/
116	exp obesity/su
117	113 or 114 or 115 or 116
118	112 not 117
119	limit 118 to (english language and humans)
120	limit 119 to ("all infant (birth to 23 months)" or "all child (0 to 18 years)" or "newborn infant (birth to 1 month)" or "infant (1 to 23 months)" or "preschool child (2 to 5 years)" or "child (6 to 12 years)")
121	119 not 120
122	(editorial or comment or letter).pt.
123	121 not 122
124	limit 123 to ed=20091208-20120530
**125**	**limit 123 to ed=20091208-20121031**

Figure S1. PRISMA flow diagram of review process

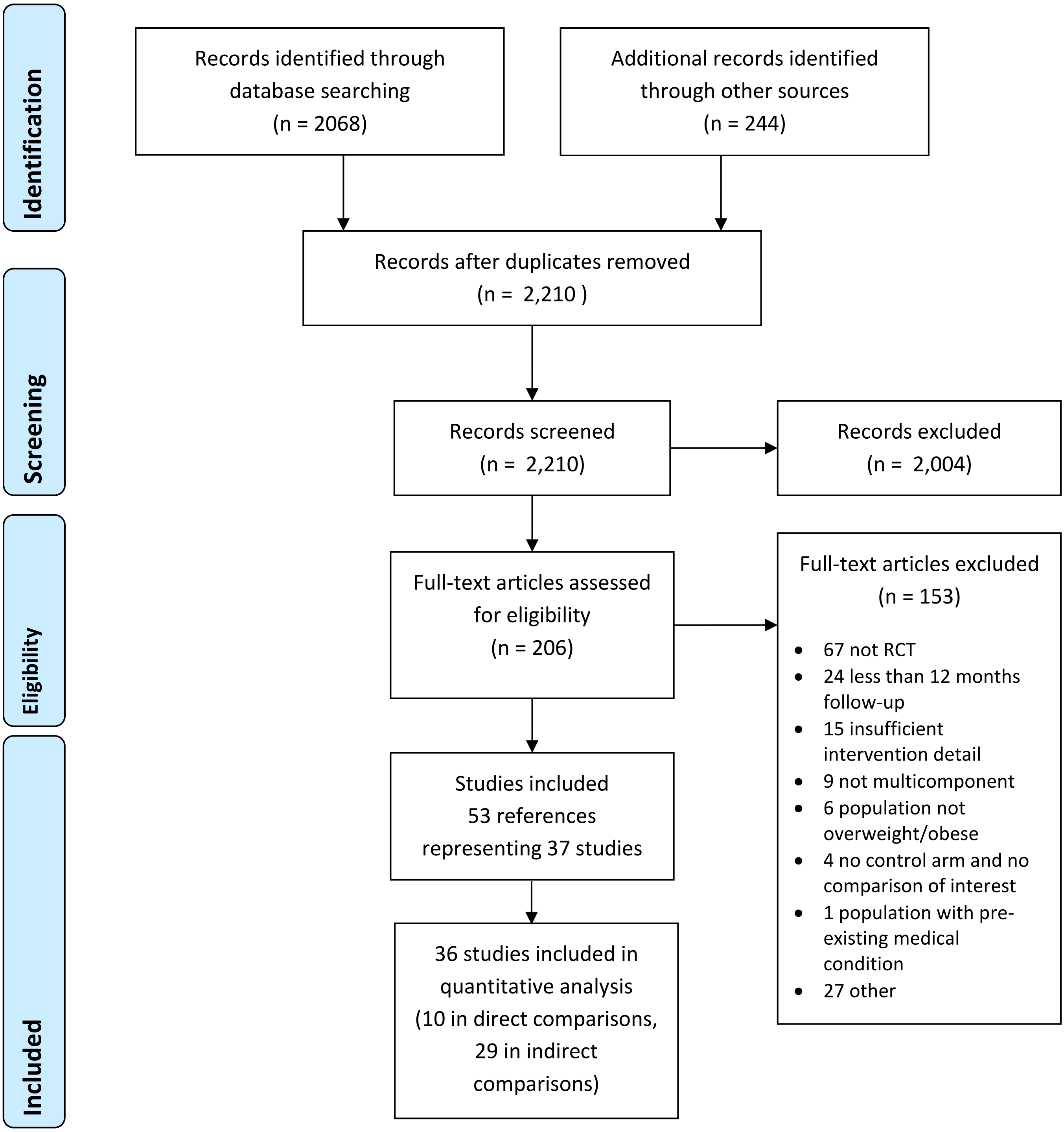

Table S1. Characteristics of included studies

Study ID, country, and total n	Intervention format[8]	Diet prescription	Weekly physical activity target[9]	Weekly weight loss target (kg)	Analyses
Appel 2011 [17]<br>*Total n:* 415<br>USA	Group and individual (intervention 1); individual (intervention 2)<br>*Mode of delivery:* Phone, web, in-person (intervention 1); phone and web only (intervention 2)<br>*Number of sessions:* 61; *Duration:* 24 months	Reduced energy diet (DASH), energy intake dependent on weight	Moderate intensity physical activity, 180 mins/wk, >10 minutes/session	NS	Intervention versus control<br>Remote versus in person support
Bertz 2012 [18]<br>*Total n:* 68<br>Sweden	Individual<br>*Mode of delivery:* in-person<br>*Number of sessions:* 2; *Duration:* 12 months	Energy restriction (deficit of 2 090kJ (500kcal)/day)	45 min brisk walking 6 days/wk	1	Intervention versus control
Dale 2009 [19]<br>*Total n:* 79<br>New Zealand	Group and individual<br>*Mode of delivery:* phone and in-person<br>*Number of sessions:* 36; *Duration:* 4 months	Macronutrient balance with some energy restriction, diets individually prescribed	30 mins vigorous activity 5 days/wk at 80-90% of age predicted maximum heart rate	NS	Intervention versus control
DPP [20]<br>*Total n:* 2161<br>USA	Group and individual<br>*Mode of delivery:* phone and in-person<br>*Number of sessions:* NR; *Duration:* NR	Reduction in dietary fat intake to <25% of energy; energy goal added if weight loss did not occur with fat restriction only	150 mins moderate intensity per week (min. 3 sessions)	0.9	Intervention versus control
Eriksson 2009 [21]<br>*Total n:* 151<br>Sweden	Group<br>*Mode of delivery:* in-person<br>*Number of sessions:* 53; *Duration:* 36 months	Reduced energy low fat diet, no target calories	Daily physical activity, 1hr sessions 3x wk	NS	Intervention versus control
Fitzgibbon 2010 [22]<br>*Total n:* 213<br>USA	Group and individual<br>*Mode of delivery:* in-person and phone<br>*Number of sessions:* 134; *Duration:* 18 months	Reduced energy and reduced fat diet, reduction based on individual	Moderate to high intensity physical activity, 30-40 mins 3-4x week, plus goal of >10,000 steps/day	NS	Intervention versus control
Foster-Schubert 2012 [23]<br>*Total n:* 439<br>USA	Group and individual<br>*Mode of delivery:* Phone, web, in-person<br>*Number of sessions:* 194; *Duration:* 12 months	Reduced energy and low fat, based on baseline weight	Moderate to high intensity physical activity, 45 mins 5x wk	NS	Intervention versus control

[8] Where more than one intervention arm, characteristics that differ by intervention arm identified as such
[9] The majority of programmes increased their physical activity targets incrementally. Details reported here are for the target at programme end. Mins: minutes; NR: not reported; wk: week.

Study ID, country, and total n	Intervention format[8]	Diet prescription	Weekly physical activity target[9]	Weekly weight loss target (kg)	Analyses
Hersey 2012 [24]<br>*Total n:* 1755<br>USA	Individual<br>*Mode of delivery:* web (intervention 1); web and phone (intervention 2)<br>*Number of sessions:* not specified (intervention 1); 39 (intervention 2)<br>*Duration:* 18 months	No specific diet, general advice encouraged reduction in energy and healthy eating	NR	NS	Intervention versus control<br>More versus less contact
Heshka 2006 [25]<br>*Total n:* 433<br>USA	Group<br>*Mode of delivery:* in-person and web<br>*Number of sessions:* 104; *Duration:* 24 months	Energy restricted balanced diet using a points system (Weight Watchers)	30 mins moderate intensity aerobic activity on 5+ /wk with 2+ resistance exercise sessions/wk	2	Intervention versus control
Jebb 2011 [26]<br>*Total n:* 772<br>UK, Germany and Australia	Group<br>*Mode of delivery:* phone, web, and in-person<br>*Number of sessions:* 52; *Duration:* 12 months	As per Heshka 2006	As per Heshka 2006	1	Intervention versus control
Jeffery 1995 [10]<br>*Total n:* 202<br>USA	Group<br>*Mode of delivery:* in-person<br>*Number of sessions:* 33; *Duration:* 18 months	Reduced energy diet, based on initial body weight	Moderate intensity physical activity 5x/week	1	Intervention versus control
Jeffery 1998 [27]<br>*Total n:* 196<br>USA	Group<br>*Mode of delivery:* in-person<br>*Number of sessions:* 36 (+ supervised walking 3x week in supervised arms); *Duration*: 18 months	Low-fat, energy restricted diet (based on initial body weight)	Moderate intensity physical activity incremental to 4 186kJ (1 000kcal)/week expenditure	NS	Supervised versus recommended physical activity
Jolly 2011 [28]<br>*Total n:* 640<br>UK	Group (commercial arms and Counterweight); individual (GP and pharmacy arms)<br>*Mode of delivery:* in-person<br>*Number of sessions:* 12; *Duration:* 3 months	Weight Watchers: as per Heshka 2006; Slimming World: Low fat low energy density; Rosemary Conley: Reduced energy low fat, energy targets based on individual; NHS Size Down: Reduced energy low fat diet based on Eatwell plate; GP/Pharmacist: As per NHS Size Down	Weight Watchers: as per Heshka 2006; Slimming World: 10x15 mins moderate activity or 5x30 mins weekly; Rosemary Conley: NR, but includes one 45min session/wk; NHS Size Down: no specific target; GP/Pharmacist: NS	Weight Watchers: 1 Slimming World: NS Rosemary Conley: 1.5; NHS Size Down: NS; GP/Pharmacist: 1	Intervention versus control
Kuller 2012 [29]<br>*Total n:* 508<br>USA	Group<br>*Mode of delivery:* in-person<br>*Number of sessions:* 64; *Duration:* 36 months	Energy and fat reduction (based on baseline weight)	Moderate intensity physical activity 240 mins/wk	NS	Intervention versus control

Study ID, country, and total n	Intervention format[8]	Diet prescription	Weekly physical activity target[9]	Weekly weight loss target (kg)	Analyses
Kumanyika 2012 [30]<br>*Total n:* 261<br>USA	Individual<br>*Mode of delivery:* in-person<br>*Number of sessions:* 17 (intervention 1); 3 (intervention 2)<br>*Duration: 12 months*	Reduced energy low fat diet	30 mins moderate intensity 5 days/wk	NS	More vs less contact
Lindstrom 2003 [31]<br>*Total n:* 522<br>Finland	Group and individual<br>*Mode of delivery:* phone and in-person<br>*Number of sessions:* 15; *Duration:* 36 months	Low fat diet (<30% kcal from fat)	30 mins moderate intensity daily	1	Intervention versus control
Logue 2005 [32]<br>*Total n:* 665<br>USA	Individual<br>*Mode of delivery:* in-person (intervention 1); in-person and phone (intervention 2)<br>*Number of sessions:* 4 (intervention 1); 28 (intervention 2)<br>*Duration: 24 months*	Energy restriction by reduced fat, eating more fruits & vegetables and smaller portions	NR	NS	More versus less contact
Mensink 2003 [33]<br>*Total n:* 114<br>Netherlands	Individual<br>*Mode of delivery:* in-person<br>*Number of sessions:* 216; *Duration:* 24 months	Fat and carbohydrate restriction based on Dutch Nutrition Council guidelines	30 mins moderate intensity 5x wk	NS	Intervention versus control
Micco 2007 [34]<br>*Total n:* 123<br>USA	Group<br>*Mode of delivery:* web only (intervention 1); web and in-person (intervention 2)<br>*Number of sessions:* 39; *Duration: 12 months*	Energy restriction based on baseline body weight	Moderate intensity 5x wk	0.9	In-person versus remote contact only
Morgan 2011 [35]<br>*Total n:* 65<br>Australia	Group and individual<br>*Mode of delivery:* in-person and web<br>*Number of sessions:* 8; *Duration:* 3 months	Reduced energy diet, based on baseline body weight	30 mins/day moderate to high intensity	NS	Intervention versus control
Munsch 2003 [36]<br>*Total n:* 122<br>Switzerland	Group<br>*Mode of delivery:* in-person<br>*Number of sessions:* 16; *Duration:* 4 months	Balanced diet, fat intake target 20g per day	15 mins daily	NS	Intervention versus control
Nanchahal 2012 [37]<br>*Total n:* 381<br>UK	Group<br>*Mode of delivery:* in-person<br>*Number of sessions:* 14; *Duration:* 8 months	Energy reduced diet based on the Eatwell plate (based on baseline body weight)	NR	1	Intervention versus control
Patrick 2011 [38]<br>*Total n:* 441<br>USA	Group and individual<br>*Mode of delivery:* web<br>*Number of sessions:* 52; *Duration:* 12 months	Balanced diet with emphasis on increasing fruit and vegetable intake	10,000 steps 5x wk and strength training 2x wk	NS	Intervention versus control
Penn 2009 [39]<br>*Total n:* 102<br>UK	Group and individual<br>*Mode of delivery:* in-person<br>*Number of sessions:* 20; *Duration:* 12 months	Low fat weight loss diet, no specific target	30 mins/day moderate intensity	NS	Intervention versus control

Study ID, country, and total n	Intervention format[8]	Diet prescription	Weekly physical activity target[9]	Weekly weight loss target (kg)	Analyses
Rejeski 2011 [40]<br>*Total n:* 288<br>USA	Group and individual<br>*Mode of delivery:* in-person and phone<br>*Number of sessions:* 48; *Duration:* 18 months	Reduced energy diet (based on baseline weight)	30-45 mins moderate intensity physical activity 5x wk	0.3	Intervention versus control
Rock 2010 [41]<br>*Total n:* 442<br>USA	Individual<br>*Mode of delivery:* Phone, web, in-person (intervention 1); phone and web (intervention 2)<br>*Number of sessions:* 104; *Duration:* 24 months	Low fat and reduced energy (based on baseline weight)	30 mins 5 or more times a week	0.9	Intervention versus control<br>In person versus remote contact only
Ross 2012 [42]<br>*Total n:* 490<br>Canada	Individual<br>*Mode of delivery:* in-person<br>*Number of sessions:* 33; *Duration:* 24 months	Mediterranean diet	45-60min moderate intensity daily	0.5	Intervention versus control
Saito 2011 [43]<br>*Total n:* 641<br>Japan	Individual<br>*Mode of delivery:* in-person<br>*Number of sessions:* 9-11 (intervention 1); 4 (intervention 2)<br>*Duration: 36 months*	Low fat diet	10,000 steps daily	NS	More versus less contact
Seligman 2011 [44]<br>*Total n:* 76<br>Brazil	Individual<br>*Mode of delivery:* in-person<br>*Number of sessions:* 7; *Duration: 3 months*	Low carb groups: high protein low carbohydrate; Low fat group: low fat diet, energy restricted to approx 8 800kJ (2 100kcal/day	Supervised: 40 mins vigorous intensity 3x week, 1 hour walking 4x wk; Recommended 10,000 steps or 1 hr walking/day	NS	Supervised versus recommended physical activity
Silva 2010 [45]<br>*Total n:* 239<br>Portugal	Group<br>*Mode of delivery:* in-person<br>*Number of sessions:* 30; *Duration:* 12 months	Reduced energy diet (reduction of daily caloric intake approx. 1 470kJ (350kcal)/day)	NR	NS	Intervention versus control
Stevens 1993 [46]<br>*Total n:* 564<br>USA	Group and individual<br>*Mode of delivery:* Phone, web, in-person<br>*Number of sessions:* 45; *Duration:* 18 months	Reduced energy diet, calculated individually	30-45 mins moderate intensity 4-5x wk	0.9	Intervention versus control
Stevens 2001 [47]<br>*Total n:* 1191<br>USA	Group and individual<br>*Mode of delivery:* in-person, phone, fax, post<br>*Number of sessions:* 47; *Duration:* 36 months	As per Stevens 1993	As per Stevens 1993	0.9	Intervention versus control
Tate 2003 [48]<br>*Total n:* 92<br>USA	Individual<br>*Mode of delivery:* web<br>*Number of sessions:* not applicable (intervention 1); 64 (intervention 2); *Duration: 12 months*	Energy intake of 5 020-6 280kJ (1 200-1 500kcal)/day, <20% of total energy intake from fat	Energy expenditure 4 190kJ (1000kcal)/week	NS	More versus less contact
Vermunt 2011 [49]<br>*Total n:* 925<br>Netherlands	Individual<br>*Mode of delivery:* in-person<br>*Number of sessions:* 17; *Duration:* 36 months	Low fat, reduced energy, high fibre diet	30 mins moderate to high intensity 5x wk	NS	Intervention versus control

**Study ID, country, and total n**	**Intervention format[8]**	**Diet prescription**	**Weekly physical activity target[9]**	**Weekly weight loss target (kg)**	**Analyses**
Villareal 2011 [50]<br>*Total n:* 107<br>USA	Group<br>*Mode of delivery:* in-person<br>*Number of sessions:* 208; *Duration:* 12 months	Energy restriction of 2 090-3 140kJ (500-750kcal) per day	90 mins moderate to high intensity 3x wk	NS	Intervention versus control
Vissers 2010 [51]<br>*Total n:* 79<br>Belgium	Individual<br>*Mode of delivery:* in-person<br>*Number of sessions:* 12; *Duration:* 12 months	Hypocaloric diet calculated on an individual level	Intervention 1: Aerobic interval training + general muscle strengthening 3x/wk; Intervention 2: Whole body vibration 3x/wk	NS	Intervention versus control
Wadden 2011 [52]<br>*Total n:* 261<br>USA	Individual<br>*Mode of delivery:* phone and in-person<br>*Number of sessions:* 25; *Duration:* 24 months	Energy restriction based on baseline weight	30 mins moderate intensity 6x wk	NS	Intervention versus control

Table S2. Risk of bias in included studies[10]

Study ID	Random sequence generation	Allocation conceal-ment	Attrition	Selective reporting	Comments
Appel 2011 [16]	LOW	LOW	LOW	LOW	
Bertz 2012 [17]	LOW	UNCLEAR	LOW	LOW	
Dale 2009 [18]	UNCLEAR	UNCLEAR	LOW	LOW	
DPP [19]	LOW	LOW	LOW	LOW	
Eriksson 2009 [20]	LOW	LOW	LOW	HIGH	BMI slightly higher in intervention group but unlikely to affect results. 6 and 36m weight measured but not reported
Fitzgibbon 2010 [21]	LOW	LOW	LOW	LOW	
Foster-Schubert 2012 [22]	LOW	LOW	LOW	LOW	
Hersey 2012 [23]	UNCLEAR	UNCLEAR	HIGH	LOW	Follow up <50% at 12 months
Heshka 2006 [24]	LOW	LOW	LOW	LOW	
Jebb 2011 [25]	LOW	LOW	LOW	LOW	
Jeffery 1995 [26]	UNCLEAR	UNCLEAR	UNCLEAR	LOW	
Jeffery 1998 [27]	UNCLEAR	UNCLEAR	LOW	HIGH	Diet outcomes and perceived barriers measured but not reported at later follow-up points
Jolly 2011 [28]	LOW	LOW	HIGH	LOW	>20% difference between arms in loss to follow up at 12m
Kuller 2012 [29]	LOW	LOW	LOW	LOW	
Kumanyika 2012 [30]	LOW	UNCLEAR	LOW	LOW	
Lindstrom 2003 [31]	LOW	LOW	LOW	LOW	
Logue 2005 [32]	LOW	LOW	LOW	LOW	
Mensink 2003 [33]	LOW	HIGH	LOW	LOW	
Micco 2007 [34]	UNCLEAR	UNCLEAR	LOW	LOW	
Morgan 2011 [35]	LOW	LOW	LOW	LOW	
Munsch 2003 [36]	HIGH	HIGH	HIGH	LOW	Those recruited from GP randomised within two GP groups. Those recruited in clinic stayed in clinic. Those recruited via newspaper unclear. Dropout higher in clinic BASEL group.
Nanchahal 2012 [37]	LOW	LOW	LOW	HIGH	Psychological variables measured but not reported
Patrick 2011 [38]	LOW	LOW	LOW	LOW	

[10] Where ‘low’ indicates low risk of bias in that domain, ‘unclear’ indicates insufficient information with which to judge, and ‘high’ indicates high risk of bias in that domain

Study ID	Random sequence generation	Allocation conceal-ment	Attrition	Selective reporting	Comments
Penn 2009 [39]	LOW	UNCLEAR	LOW	HIGH	Authors measured waist circumference and weight annually and did not report it as the differences were not significant
Rejeski 2011 [40]	UNCLEAR	UNCLEAR	LOW	HIGH	Authors do not report weight at 12 months although the article suggests this would have been measured.
Rock 2010 [41]	LOW	LOW	LOW	LOW	
Ross 2012 [42]	LOW	UNCLEAR	LOW	LOW	Allocation method not specified but conducted by data manager
Saito 2011 [43]	LOW	LOW	LOW	HIGH	Weight change measured at 12, 24 and 36m but only reported at 12m; however authors provided
Seligman 2011 [44]	LOW	LOW	LOW	LOW	
Silva 2010 [45]	LOW	LOW	LOW	HIGH	Data on BMI and weight change missing at some follow-up points
Stevens 1993 [46]	UNCLEAR	LOW	LOW	LOW	
Stevens 2001 [47]	UNCLEAR	LOW	LOW	HIGH	BMI not reported at a number of time points
Tate 2003 [48]	LOW	UNCLEAR	LOW	LOW	
Vermunt 2011 [49]	HIGH	HIGH	LOW	HIGH	Allocation to intervention and control was alternate and known to GP prior to enrolment. Weight data missing at a number of time points
Villareal 2011 [50]	LOW	UNCLEAR	LOW	LOW	
Vissers 2010 [51]	UNCLEAR	UNCLEAR	HIGH	LOW	Uneven dropouts between arms
Wadden 2011 [52]	LOW	LOW	LOW	LOW	

References (complete list)

1. LeBlanc ES, O'Connor E, Whitlock EP *et al*. Effectiveness of primary care-relevant treatments for obesity in adults: A systematic evidence review for the US Preventive Services Task Force. *Annals of Internal Medicine* 2011; **155**(7)**:** 434-U71.
2. Loveman E, Frampton GK, Shepherd J *et al.* The clinical effectiveness and cost-effectiveness of long-term weight management schemes for adults: a systematic review. *Health Technology Assessment* 2011; **15**(2)**:** 1-182.
3. Hartmann-Boyce J, Johns D, Aveyard P *et al. Managing overweight and obese adults: The clinical effectiveness of long-term weight management schemes for adults*. University of Oxford: Oxford, 2013. http://www.nice.org.uk/guidance/index.jsp?action=download&o=65478
4. Abraham C, Michie S. A taxonomy of behavior change techniques used in interventions. *Health Psychology*2008; **27**(3)**:** 379-87.
5. Michie S, Ashford S, Sniehotta FF, Dombrowski SU, Bishop A, French DP. A refined taxonomy of behaviour change techniques to help people change their physical activity and healthy eating behaviours: the CALO-RE taxonomy. *Psychology & Health* 2011; **26**(11)**:** 1479-98.
6. Michie S, Richardson M, Johnston M *et al.* The Behavior Change Technique Taxonomy (v1) of 93 hierarchically clustered techniques: Building an international consensus for the reporting of behavior change interventions. *Annals of Behavioral Medicine* 2013; **46**(1)**:** 81-95.
7. NHS Centre for Reviews and Dissemination. *Systematic reviews: CRD's guidance for undertaking reviews in healthcare*. Centre for Reviews and Dissemination: York, UK, 2008.
8. Kaiser KA, Affuso O, Beasley TM, Allison DB. Getting carried away: a note showing baseline observation carried forward (BOCF) results can be calculated from published complete-cases results. *International Journal of Obesity* 2012; **36**(6)**:** 886-9.
9. Jeffery RW, Wing RW. Long-term effects of interventions for weight loss using food provision and monetary incentives. *Journal of Consulting and Clinical Psychology* 1995; **63** (5): 793-6.
10. Review Manager (RevMan) Version 5.2 ed. Copenhagen: The Nordic Cochrane Centre, The Cochrane Collaboration, 2012.
11. Higgins JP, Thompson SG, Deeks JJ, Altman DG. Measuring inconsistency in meta-analyses. *BMJ* 2003; **327**(7414)**:** 557-60.
12. Riley RD, Higgins JPT, Deeks JJ. Interpretation of random effects meta-analyses. *BMJ* 2011; **342**: d549.
13. Dombrowski SU, Sniehotta FF, Avenell A, Johnston M, MacLennan G, Araújo-Soares V. Identifying active ingredients in complex behavioural interventions for obese adults with obesity-related co-morbidities or additional risk factors for co-morbidities: a systematic review. *Health Psychology Review* 2012; **6**(1)**:** 7-32.
14. Olander EK, Fletcher H, Williams S, Atkinson L, Turner A, French DP. What are the most effective techniques in changing obese individuals' physical activity self-efficacy and behaviour: a systematic review and meta-analysis. *The International Journal of Behavioral Nutrition and Physical Activity* 2013; **10:** 29.
15. Michie S, Abraham C, Whittington C, McAteer J, Gupta S. Effective techniques in healthy eating and physical activity interventions: a meta-regression. *Health Psychology* 2009; **28**(6)**:** 690-701.
16. Michie S, Hardeman W, Fanshawe T, Prevost AT, Taylor L, Kinmonth AL. Investigating theoretical explanations for behaviour change: The case study of ProActive. *Psychology & Health* 2007; **23**(1)**:** 25-39.
17. Appel LJ, Clark JM, Yeh HC *et al.* Comparative effectiveness of weight-loss interventions in clinical practice. *New England Journal of Medicine* 2011; **365**(21)**:** 1959-1968.
18. Bertz F, Brekke HK, Ellegard L, Rasmussen KM, Wennergren M, Winkvist A. Diet and exercise weight-loss trial in lactating overweight and obese women. *American Journal of Clinical Nutrition* 2012; **96**(4)**:** 698-705.
19. Dale KS, Mann JI, McAuley KA, Williams SM, Farmer VL. Sustainability of lifestyle changes following an intensive lifestyle intervention in insulin resistant adults: Follow-up at 2-years. *Asia Pacific Journal of Clinical Nutrition* 2009; **18**(1)**:** 114-120.
20. Knowler WC, Barrett-Connor E, Fowler SE *et al.* Reduction in the incidence of type 2 diabetes with lifestyle intervention or metformin. *New England Journal of Medicine* 2002; **346**(6)**:** 393-403.

21. Eriksson MK, Franks PW, Eliasson M. A 3-year randomized trial of lifestyle intervention for cardiovascular risk reduction in the primary care setting: The Swedish Bjorknas study. *Plos One* 2009; **4**(4)**:** e5195.
22. Fitzgibbon ML, Stolley MR, Schiffer L, Sharp LK, Singh V, Dyer A. Obesity reduction black intervention trial (ORBIT): 18-month results. *Obesity* 2010; **18**(12)**:** 2317-2325.
23. Foster-Schubert KE, Alfano CM, Duggan CR *et al.* Effect of diet and exercise, alone or combined, on weight and body composition in overweight-to-obese postmenopausal women. *Obesity* 2012; **20**(8)**:** 1628-1638.
24. Hersey JC, Khavjou O, Strange LB *et al.* The efficacy and cost-effectiveness of a community weight management intervention: a randomized controlled trial of the health weight management demonstration. *Preventive Medicine* 2012; **54**(1)**:** 42-49.
25. Heshka S, Anderson JW, Atkinson RL *et al* . Weight loss with self-help compared with a structured commercial program: a randomised trial. *JAMA* 2003; **289** (14): 1792-8.
26. Jebb SA, Ahern AL, Olson AD *et al.* Primary care referral to a commercial provider for weight loss treatment versus standard care: a randomised controlled trial. *Lancet* 2011; **378**(9801)**:** 1485-1492.
27. Jeffery RW, Wing R, Thorson C, Burton LR. Use of personal trainers and financial incentives to increase exercise in a behavioural weight loss program. *Journal of Consulting and Clinical Psychiatry* 1998; **66**(5): 777-783.
28. Jolly K, Lewis A, Beach J *et al.* Comparison of range of commercial or primary care led weight reduction programmes with minimal intervention control for weight loss in obesity: Lighten Up randomised controlled trial. *BMJ* 2011; **343:** d6500.
29. Kuller LH, Pettee Gabriel KK, Kinzel LS *et al.* The Women on the Move Through Activity and Nutrition (WOMAN) study: final 48-month results. *Obesity* 2012; **20**(3)**:** 636-643.
30. Kumanyika SK, Fassbender JE, Sarwer DB *et al.* One-year results of the Think Health! study of weight management in primary care practices. *Obesity* 2012; **20**(6)**:** 1249-1257.
31. Lindstrom J, Louheranta A, Mannelin M *et al*. The Finnish Diabetes Prevention Study (DPS): Lifestyle intervention and 3-year results on diet and physical activity. *Diabetes Care* 2003; **26**: 3230-3236.
32. Logue E SK, Jarjoura D, Smucker W, Baughman K, Capers C. Transtheoretical model-chronic disease care for obesity in primary care: a randomised trial. *Obesity Research* 2005; **13**:917-927.
33. Mensink M, Blaak EE, Corpeleijn E *et al*. Lifestyle interventions according to general recommendations improves glucose tolerance. *Obesity Research* 2003; **11**(12): 1588-1596.
34. Micco N, Gold B, Buzzell P, Leonard H, Pintauro S, Harvey-Berino J . Minimal in-person support as an adjunct to internet obesity treatment. *Annals of Behavioral Medicine* 2007: **33**(1): 49-56.
35. Morgan PJ, Lubans DR, Collins CE, Warren JM, Callister R. 12-month outcomes and process evaluation of the SHED-IT RCT: an internet-based weight loss program targeting men. *Obesity* 2011; **19**(1)**:** 142-151.
36. Munsch S, Biedert E, Keller U. Evaluation of a lifestyle change programme for the treatment of obesity in general practice. *Swiss Medical Weekly* 2003*;***133**(9-10):148-154.
37. Nanchahal K, Power T, Holdsworth E *et al.* A pragmatic randomised controlled trial in primary care of the Camden Weight Loss (CAMWEL) programme. *BMJ Open* 2012; **2**(3).
38. Patrick K, Calfas KJ, Norman GJ *et al.* Outcomes of a 12-month web-based intervention for overweight and obese men. *Annals of Behavioral Medicine* 2011; **42**(3)**:** 391-401.
39. Penn L, White M, Oldroyd J, Walker M, Alberti KG, Mathers JC. Prevention of type 2 diabetes in adults with impaired glucose tolerance: the European Diabetes Prevention RCT in Newcastle upon Tyne, UK. *BMC Public Health* 2009; **9**: 342.
40. Rejeski WJ, Brubaker PH, Goff DC Jr. *et al.* Translating weight loss and physical activity programs into the community to preserve mobility in older, obese adults in poor cardiovascular health. *Archives of Internal Medicine* 2011; **171**(10)**:** 880-886.
41. Rock CL, Flatt SW, Sherwood NE, Karanja N, Pakiz B, Thomson CA. Effect of a free prepared meal and incentivized weight loss program on weight loss and weight loss maintenance in obese and overweight women: a randomized controlled trial. *JAMA* 2010; **304**(16)**:** 1803-1810.
42. Ross R, Lam M, Blair SN *et al.* Trial of prevention and reduction of obesity through active living in clinical settings: a randomized controlled trial. *Archives of Internal Medicine* 2012; **172**(5)**:** 414-424.
43. Saito T, Watanabe M, Nishida J *et al.* Lifestyle modification and prevention of type 2 diabetes in overweight Japanese with impaired fasting glucose levels: a randomized controlled trial. *Archives of Internal Medicine* 2011; **171**(15)**:** 1352-1360.

44. Seligman BGS, Polanczyk CA, Santos ASB *et al.* Intensive practical lifestyle intervention improves endothelial function in metabolic syndrome independent of weight loss: a randomized controlled trial. *Metabolism-Clinical and Experimental* 2011; **60**(12)**:** 1736-1740.
45. Silva MN, Vieira PN, Coutinho SR *et al.* Using self-determination theory to promote physical activity and weight control: a randomized controlled trial in women. *Journal of Behavioral Medicine* 2010; **33**(2)**:** 110-122.
46. Stevens VJ, Corrigan SA, Obarzanek E, Bernauer E *et al*. Weight loss intervention in Phase 1 of the trials of hypertension prevention. *Archives of Internal Medicine* 1993; **153**: 849-858.
47. Stevens VJ, Obarzanek E, Cook NR *et al*. Long-term weight loss and changes in blood pressure: Results of the trials of hypertension prevention, phase II. *Annals of Internal Medicine* 2001; **134**(1): 1-11.
48. Tate DF, Jeffery RW, Sherwood NE, Wing RR. Long-term weight losses associated with prescription of higher physical activity goals. Are higher levels of physical activity protective against weight regain? *American Journal of Clinical Nutrition* 2007; **85**(4): 954-9.
49. Vermunt PW, Milder IE, Wielaard F, de Vries JH, van Oers HA, Westert GP. Lifestyle counseling for type 2 diabetes risk reduction in Dutch primary care: results of the APHRODITE study after 0.5 and 1.5 years. *Diabetes Care* 2011; **34**(9)**:** 1919-1925.
50. Villareal DT, Chode S, Parimi N *et al.* Weight loss, exercise, or both and physical function in obese older adults. *New England Journal of Medicine* 2011; **364**(13)**:** 1218-1229.
51. Vissers D, Verrijken A, Mertens I *et al.* Effect of long-term whole body vibration training on visceral adipose tissue: a preliminary report. *Obesity Facts* 2010; **3**(2)**:** 93-100.
52. Wadden TA, Volger S, Sarwer DB *et al*. A two-year randomized trial of obesity treatment in primary care practice. *New England Journal of Medicine* 2011; **365**(21): 1969-79.
